# Hypoxia sensing in resident cardiac macrophages regulates monocyte fate specification following ischemic heart injury

**DOI:** 10.1038/s44161-024-00553-6

**Published:** 2024-10-21

**Authors:** Farid F. Kadyrov, Andrew L. Koenig, Junedh M. Amrute, Hao Dun, Wenjun Li, Carla J. Weinheimer, Jessica M. Nigro, Attila Kovacs, Andrea L. Bredemeyer, Steven Yang, Shibali Das, Vinay R. Penna, Alekhya Parvathaneni, Lulu Lai, Niklas Hartmann, Benjamin J. Kopecky, Daniel Kreisel, Kory J. Lavine

**Affiliations:** 1 Center for Cardiovascular Research, Division of Cardiology, Department of Medicine, Washington University School of Medicine, Saint Louis, MO, USA.; 2 Division of Cardiothoracic Surgery, Department of Surgery, Washington University School of Medicine, Saint Louis, MO, USA.; 3 German Center for Cardiovascular Research (DZHK), Partner Site Heidelberg, Heidelberg, Germany.; 4 Department of Cardiology, Internal Medicine III, Heidelberg University Hospital, Heidelberg, Germany.; 5 Department of Pathology and Immunology, Washington University School of Medicine, Saint Louis, MO, USA.; 6 Department of Developmental Biology, Washington University School of Medicine, Saint Louis, MO, USA.

## Abstract

Myocardial infarction initiates cardiac remodeling and is central to heart failure pathogenesis. Following myocardial ischemia–reperfusion injury, monocytes enter the heart and differentiate into diverse subpopulations of macrophages. Here we show that deletion of *Hif1α*, a hypoxia response transcription factor, in resident cardiac macrophages led to increased remodeling and overrepresentation of macrophages expressing arginase 1 (*Arg1*). Arg1^+^ macrophages displayed an inflammatory gene signature and may represent an intermediate state of monocyte differentiation. Lineage tracing of Arg1^+^ macrophages revealed a monocyte differentiation trajectory consisting of multiple transcriptionally distinct states. We further showed that deletion of *Hif1α* in resident cardiac macrophages resulted in arrested progression through this trajectory and accumulation of an inflammatory intermediate state marked by persistent *Arg1* expression. Depletion of the Arg1^+^ trajectory accelerated cardiac remodeling following ischemic injury. Our findings unveil distinct trajectories of monocyte differentiation and identify hypoxia sensing as an important determinant of monocyte differentiation following myocardial infarction.

Myocardial infarction (MI) and other forms of ischemic cardiac injury initiate adverse left ventricular (LV) remodeling and are central to the pathogenesis of heart failure^1,2^. Heart failure continues to represent a life-threatening complication of MI despite the availability of effective medications that limit adverse LV remodeling^1–3^. Activation of innate immune responses and the resultant inflammation that occurs after MI may represent a promising therapeutic target to limit LV remodeling and heart failure^4^.

Macrophages represent the primary immune cell found in the heart under homeostasis. Cardiac macrophages are complex, arising from multiple lineages. The mouse and human heart contain two functionally distinct resident macrophage populations distinguished by C–C chemokine receptor 2 (CCR2) expression: embryonic-derived resident CCR2^−^ macrophages that promote tissue repair and suppress inflammation and monocyte-derived CCR2^+^ macrophages that initiate inflammatory responses^5–8^. Following MI, large numbers of Ly6C^hi^CCR2^+^ monocytes are recruited to the heart and differentiate into diverse macrophage populations that are traditionally viewed as having proinflammatory properties^9,10^. Additionally, under steady-state conditions, embryonic-derived cardiac resident macrophages can be replaced by monocyte-derived macrophages over the course of aging^11^.

Prior studies have elucidated that manipulating cardiac macrophage composition and interfering with monocyte recruitment has dramatic impacts on MI outcomes^5,6,12–14^. Depletion of CCR2^+^ macrophages before MI is sufficient to reduce neutrophil/monocyte infiltration, suppress myocardial chemokine/cytokine production and preserve LV systolic function. Conversely, removing CCR2^−^ tissue resident macrophages leads to increased peripheral leukocyte recruitment and accelerated LV remodeling. Consistent with a deleterious role for infiltrating leukocytes, inhibition of monocyte recruitment has beneficial effects in mouse models of MI^5,13–16^.

Recent single-cell RNA sequencing (scRNAseq) studies indicated that monocytes recruited to the injured heart differentiate into multiple distinct macrophage and dendritic cell-like subsets^5^. The mechanistic basis and dynamics by which monocytes differentiate into these populations are unknown. We hypothesized that environmental cues present within the infarcted heart instruct monocyte differentiation decisions. These cues may act either directly on infiltrating monocytes or indirectly through non-cell autonomous mechanisms. The existence of non-cell autonomous pathways is supported by the observation that cardiac resident macrophage depletion before MI is sufficient to alter the relative abundance of various monocyte-derived macrophage subsets^5^.

Here, we explored hypoxia sensing as an environmental cue that may influence monocyte differentiation following MI using mouse models of myocardial ischemia–reperfusion (I/R) injury. Hypoxia inducible factor-1 (HIF1) is a transcription factor that regulates the cellular response to hypoxia^17–19^. We conditionally deleted *Hif1α* in either all monocytes and macrophages, recruited monocytes and macrophages or resident macrophages. We demonstrate that *Hif1α* deletion in resident macrophages accelerated LV remodeling and led to expansion of a subpopulation of monocyte-derived macrophages marked by arginase 1 (*Arg1*) expression. Arg1^+^ macrophages represented a population that was specified early during monocyte differentiation and displayed an inflammatory gene signature. Lineage tracing demonstrated that Arg1^+^ macrophages differentiate into multiple distinct macrophage subsets including Gdf15^+,^ Trem2^+^, MHC-II^hi^ and resident-like macrophages. *Hif1α* deletion in resident macrophages arrested progression through this differentiation trajectory, and proper progression of this trajectory was protective in the context of ischemic heart injury. These findings identify distinct trajectories of monocyte-derived macrophages within the infarcted heart and identify hypoxia sensing in resident cardiac macrophages as a non-cell autonomous mediator of monocyte differentiation.

## Results

### *Hif1α* KO in monocytes/macrophages accelerates MI remodeling

**Extended Data Fig. 1 |:**
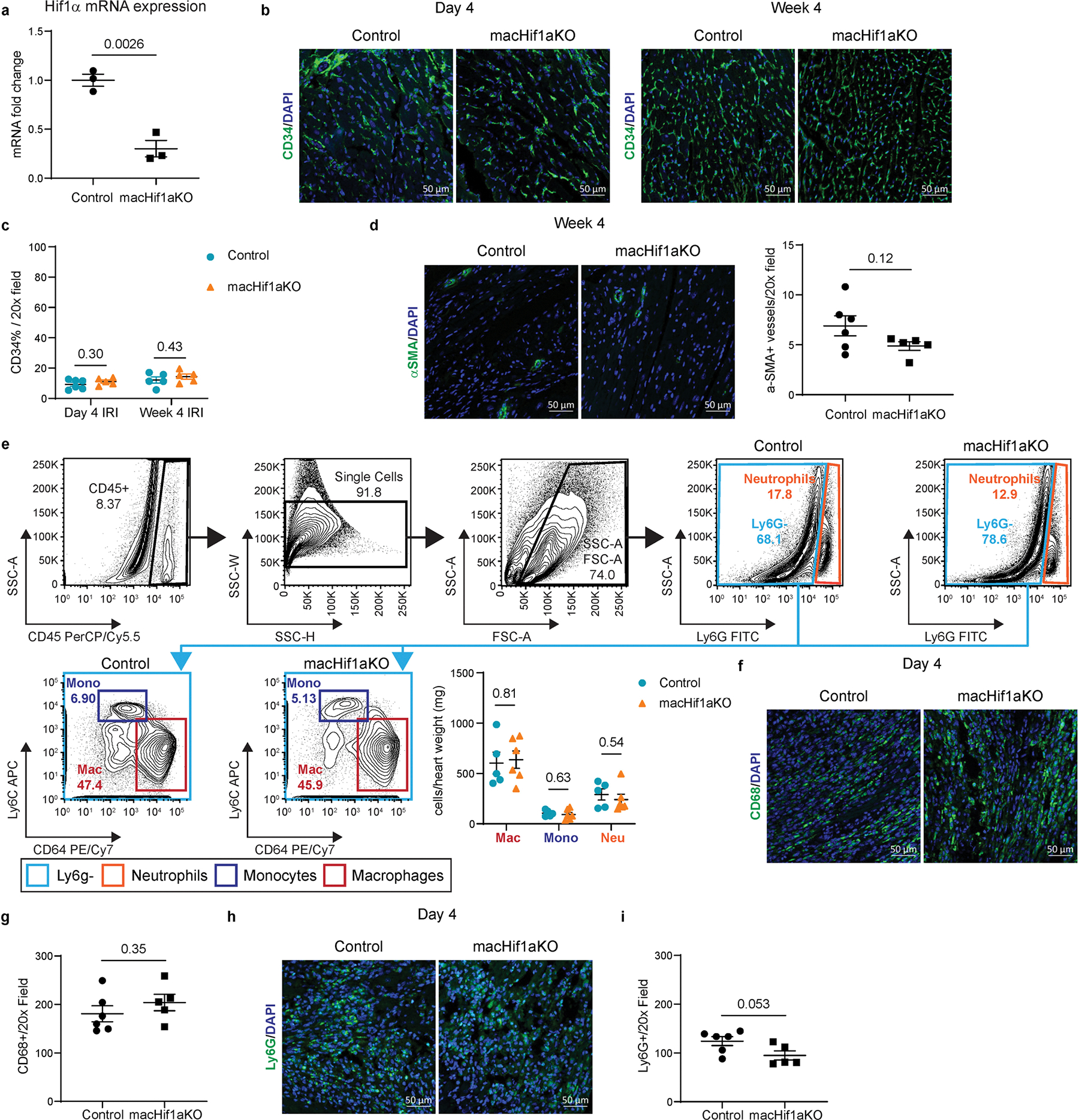
Hif1α deletion in monocytes and macrophages does not impact angiogenesis or the myeloid compartment after MI. **a**, qPCR of *Hif1α* mRNA expression in sorted monocytes and macrophages from Control and macHif1aKO mice 5 days after I/R. N = 3. **b**, Representative 20x confocal images of CD34 (green) IHC staining within the border zone of Control and macHif1aKO mice 4 days (N = 6 vs 5) and 4 weeks (N = 5) after I/R. DAPI is in blue. **c**, Quantification of b, displayed as the percentage of CD34 signal per 20x field. **d**, Representative 20x confocal images of αSMA (green) IHC in the border zone of Control and macHif1aKO mice 4 weeks after I/R. DAPI is in blue. Quantification is displayed as the total number of αSMA^+^ vessels per 20x field. N = 6 vs 5. **e**, FACS from Control and macHif1aKO mice 5 days after I/R. Quantification shows cell numbers normalized to mg of heart tissue used for FACS. N = 5 vs 6. **f**, Representative 20x confocal images of macrophage IHC within the infarct of Control and macHif1aKO mice 4 days after I/R. CD68 staining is in green, DAPI is in blue. **g**, Quantification of f displayed as the total number of CD68^+^ cells per 20x field. N = 6 vs 5. **h**, Representative 20x confocal images of neutrophil IHC within the infarct of Control and macHif1aKO mice 4 days after I/R. Ly6G staining is in green and DAPI staining is in blue. **i**, Quantification of h displayed as the total number of Ly6G^+^ cells per 20x field. N = 6 vs 5. P-values are determined using a two tailed t-test assuming equal variance. For N, each data point is an individual mouse. Data are presented as mean ± SEM.

**Fig. 1 |:**
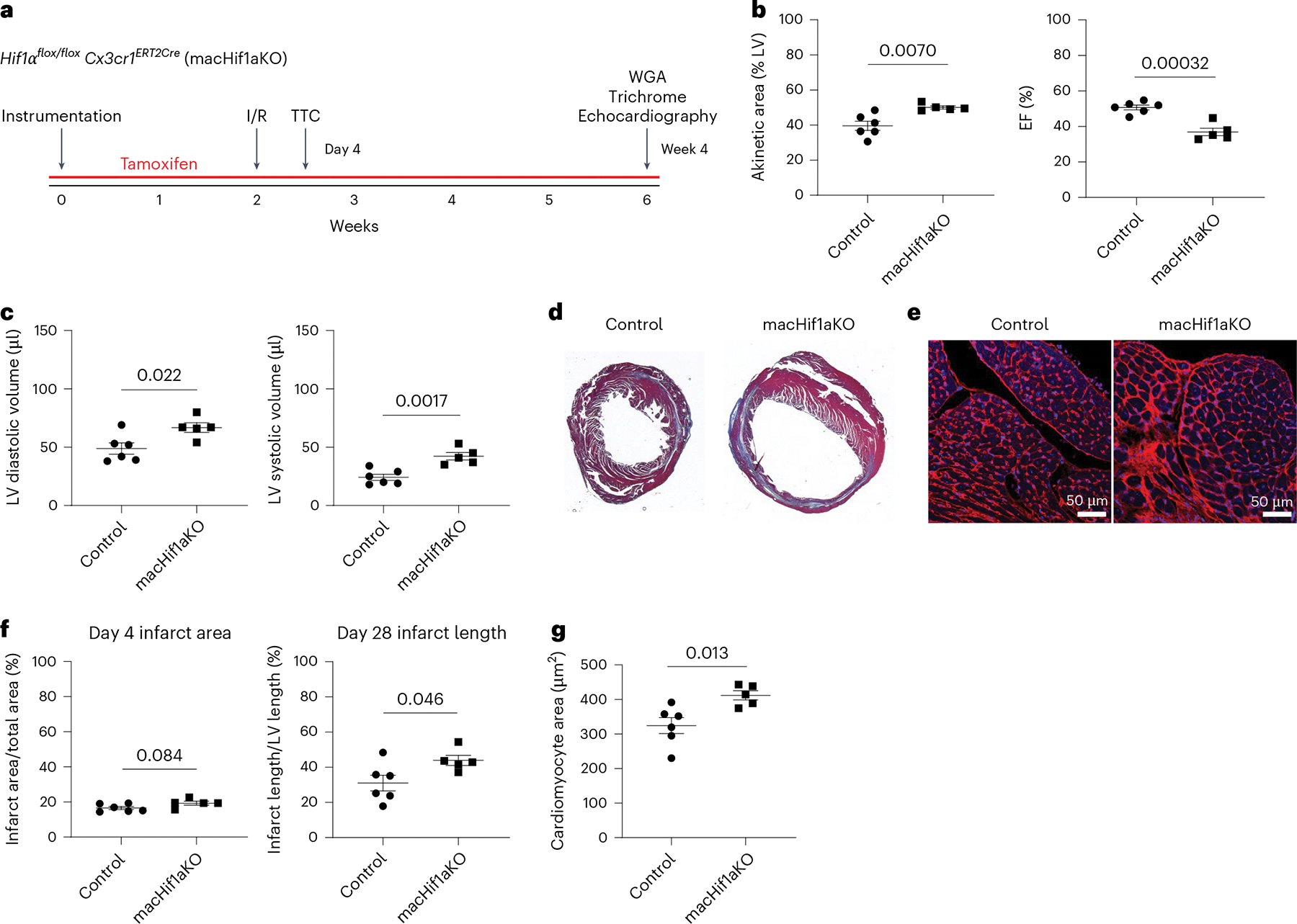
Hif1α deletion in monocytes and macrophages leads to accelerated LV remodeling and reduced cardiac function after MI. **a**, Experimental outline of the I/R injury mouse model of MI in macHif1aKO mice. Tamoxifen chow was given throughout the course of the experiment. Closed-chest I/R was performed 2 weeks after instrumentation, and echocardiography was performed 4 weeks after I/R. Tissues were collected 4 days and 4 weeks after I/R fsor analysis. **b**, Left: echocardiography quantification 4 weeks after I/R of the akinetic area as a percentage of the left ventricle. Right: a comparison of the EF between control and macHif1aKO mice. **c**, A comparison of the LV diastolic volume (left) and LV systolic volume (right) between control and macHif1aKO mice. **d**, Representative trichrome staining of control and macHif1aKO hearts 4 weeks after I/R. **e**, Representative WGA staining of the border zone of the infarct of control and macHif1aKO hearts 4 weeks after I/R. **f**, Quantification of TTC staining 4 days after I/R to determine the initial infarct area (displayed as a percentage of infarct area/total area) and quantification of trichrome staining in **d** (displayed as the length of the infarct as a percentage of the total length of the left ventricle) for control versus macHif1aKO mice. **g**, Quantification of the average cardiomyocyte area determined from WGA staining in **e** in control and macHif1aKO hearts. *P* values were determined using a two tailed *t*-test assuming equal variance. *N* = 6 versus 5 (each data point is an individual mouse). Data presented as mean ± s.e.m.

To assess the functional requirement for hypoxia sensing in monocytes and macrophages following MI, we subjected control and *Hif1α*^*flox/flox*^ *Cx3cr1*^*ERT2Cre*^ mice to closed-chest I/R injury (90 min of ischemia) to simulate reperfused MI^20,21^. Tamoxifen chow was given throughout the entirety of the experiment to induce *Hif1α* deletion (knockout (KO)) in all monocytes and macrophages (macHif1aKO) (Fig. 1a and Extended Data Fig. 1a). While many genes with unique and overlapping roles are involved in the cell’s response to hypoxia (including *Hif1α*, *Arnt*, *Epas1*, *Arnt2* and *Hif3α*), we focused on *Hif1α* as it had the highest messenger RNA expression in cardiac macrophages in several sequencing datasets of heart injury^5,15,22–25^ (Supplementary Fig. 1). Echocardiography performed 4 weeks after I/R revealed that macHif1aKO mice had increased akinetic LV area, reduced LV ejection fraction (EF) and increased LV diastolic and systolic volumes (Fig. 1b,c). Trichrome and WGA (wheat germ agglutinin) staining performed 4 weeks after I/R revealed increased infarct size and cardiomyocyte hypertrophy in macHif1aKO mice (Fig. 1d–g). TTC (triphenyl tetrazolium chloride) staining performed within 4 days after I/R showed no difference in initial infarct area between control and macHif1aKO mice, indicating that increased final infarct size in macHif1aKO mice is due to infarct expansion (Fig. 1f).

In the context of MI and heart failure, *Hif1α* is thought to contribute to expansion of the coronary vasculature within the infarct and border zones^26^. Immunostaining (IHC) for CD34 and smooth muscle actin did not reveal any reduction in vascular density in macHif1aKO hearts following I/R (Extended Data Fig. 1b–d)^27^. To assess whether *Hif1α* deletion in monocytes and macrophages alters the abundance of the myeloid cells that infiltrate the heart after MI^28^, we performed IHC and flow cytometry analysis (FACS) of control and macHif1aKO mice 4–5 days after I/R. We observed no differences in the number of monocytes or macrophages between conditions and a marginal reduction in neutrophils in macHif1aKO hearts (Extended Data Fig. 1e–i).

### *Hif1α* KO shifts monocyte and macrophage fate after MI

**Extended Data Fig. 2 |:**
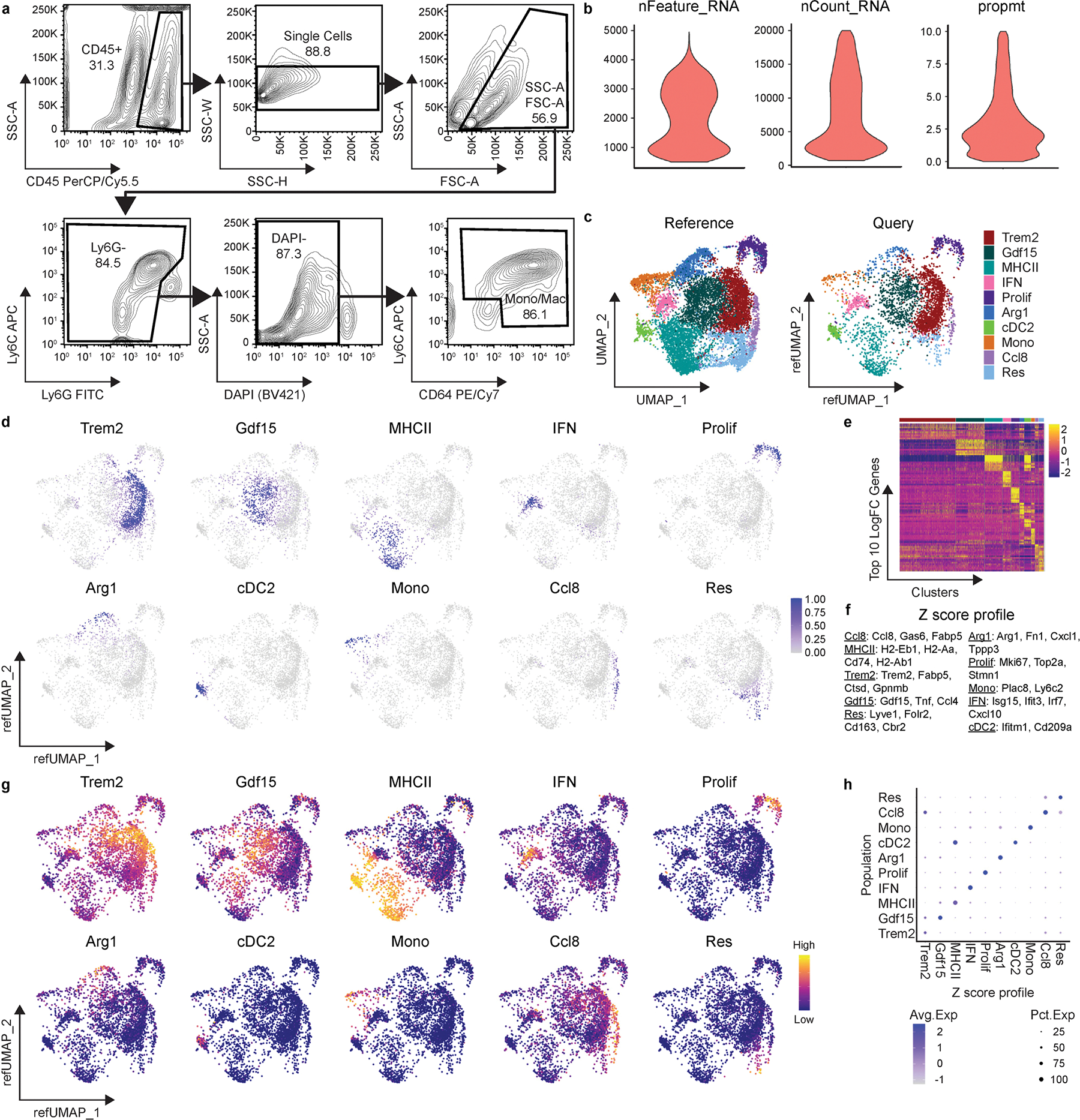
Single cell RNA sequencing and reference mapping of cardiac monocytes, macrophages, and dendritic-like cells 5 days after MI. **a**, FACS gating strategy to isolate monocytes, macrophages, and dendritic-like cells from Control and macHif1aKO mice 5 days after I/R for use in scRNAseq. Cells were gated for leukocytes (SSC-A vs CD45), single cells (SSC-W vs SSC-H), debris exclusion (SSC-A vs FSC-A), neutrophil exclusion (Ly6C vs Ly6G), live cells (SSC-A vs DAPI) and monocytes/macrophages (Ly6C vs CD64). **b**, Violin plots representing quality control of scSEQ data post quality control. Cells with greater than 500 and less than 5000 genes (nFeature_RNA), read counts less than 20,000 (nCount_RNA), and proportion of transcripts mapping to mitochondrial genes less than 10 percent (prompt) were used for downstream analysis. **c**, UMAP projection and annotations from the reference MI scSEQ data set (monocytes, macrophages, and dendritic-like cells 3, 7, 13, and 28 days after I/R) and the query dataset (Control and macHif1aKO monocytes, macrophages, and dendritic-like cells 5 days after I/R) after reference mapping in Seurat. Query dataset is plotted on a reference UMAP (refUMAP) **d**, Mapping scores of each subpopulation projected onto refUMAP of the query data set (Control and macHif1aKO). **e**, Heatmap of the top 10 upregulated genes in each subpopulation across the entire Control and macHif1aKO query data set. **f**, Z-score profile genes of each subpopulation from the reference data set. **g**, Z-scores from f projected onto refUMAP of the query data set (Control and macHif1aKO. **h**, Z-scores from f compared to each subpopulation in the query data set represented as a dot-plot.

**Fig. 2 |:**
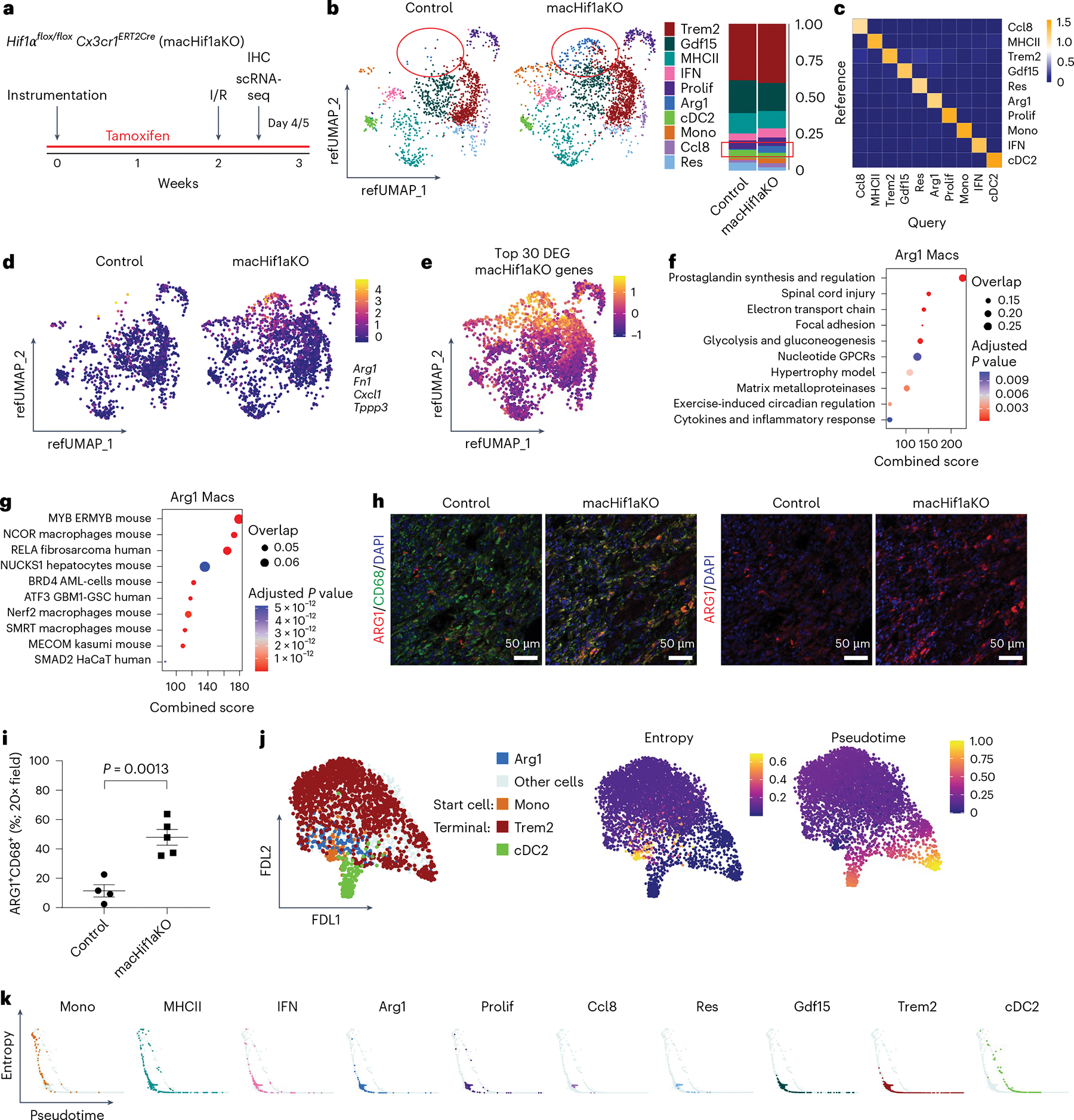
scRNAseq of monocytes, macrophages and dendritic-like cells 5 days after MI reveals overrepresentation of Arg1^+^ macrophages in macHif1aKO mice. **a**, Experimental outline of the I/R mouse model of MI in macHif1aKO mice. Tissues were collected 4 days after I/R for IHC and 5 days after I/R for scRNAseq. **b**, Annotated reference UMAP (refUMAP) of scRNAseq data and stacked bar graph representing the proportion of each cell population split between control and macHif1aKO mice. Data were mapped to a reference MI dataset of monocytes/macrophages/dendritic-like cells 3, 7, 13 and 28 days after I/R. The red circles and box highlight Arg1^+^ macrophages in each condition. Prolif, proliferating; Mono, monocytes; Res, resident. **c**, A heat map of mapping scores for each cell population in the query (control and macHif1aKO scRNAseq) plotted against the reference MI dataset. **d**, *Z*-score profile of Arg1^+^ macrophages (*Arg1*, *Fn1*, *Cxcl1* and *Tpp3*) plotted on a reference UMAP and split between conditions. **e**, *Z*-score profile of the top 30 differentially expressed genes (DEGs) upregulated in macHif1aKO mice plotted on a reference UMAP. **f**, A dot plot of the top ten upregulated pathways (Enrichr Wikipathways 2019 Mouse) in Arg1^+^ macrophages. **g**, A dot plot of the top ten ChEA 2016 EnrichR transcription factors upregulated in Arg1^+^ macrophages. **h**, Confocal images (20×) of ARG1 (red), CD68 (green) and DAPI (blue) IHC staining within the infarct zone 4 days after I/R of control and macHif1aKO mice. **i**, Quantification of IHC in **h**, displayed as the percentage of CD68^+^ cells that are ARG1^+^CD68^+^. *N* = 4 versus 5 (each data point is an individual mouse). Data are presented as mean ± s.e.m. **j**, FDL representation of Palantir analysis displaying the starting cells (Mono, orange), predicted terminal states (Trem2, red and cDC2, green), Arg1 cells (blue) and all other cells (gray). Entropy and pseudotime scores from Palantir plotted on FDL. **k**, Plots of entropy versus pseudotime from Palantir for each subpopulation. *P* values were determined using a two tailed *t*-test assuming equal variance.

Previous scRNAseq studies revealed that altering the identity of macrophages present within the heart following MI has profound effects on LV remodeling^5,15^. To evaluate whether *Hif1α* deletion in monocytes and macrophages impacts the differentiation of infiltrating monocytes following I/R, we utilized scRNAseq on cardiac monocytes, macro phages and dendritic-like cells isolated by FACS 5 days after I/R to identify transcriptionally distinct populations of monocytes and their progeny in control and macHif1aKO mice (Fig. 2a,b, Extended Data Fig. 2a,b and Supplementary Table 1). To consistently annotate cell populations with high confidence, scRNAseq data generated from control and macHif1aKO mice were mapped using label transfer^29^ to a reference dataset of myeloid cells isolated from the mouse heart 3, 7, 14 and 28 days after MI^30^ (Extended Data Fig. 2c). Reference mapping performance was validated by calculating mapping scores and plotting transcriptional signatures of reference cell populations in the query dataset (Fig. 2c and Extended Data Fig. 2d–h).

Composition analysis of monocytes, macrophages and dendritic-like cells demonstrated an overrepresentation of a macro phage population marked by *Arg1* mRNA expression in macHif1aKO mice (Fig. 2a,b), suggesting that hypoxia sensing in monocytes and/or macrophages may influence the differentiation of infiltrating monocytes. Arg1^+^ macrophages had a unique transcriptional signature with enrichment in *Arg1*, *Fn1*, *Cxcl1* and *Tppp3* mRNA expression (Fig. 2d). Differential gene expression analysis revealed that many of the genes increased in macHif1aKO monocytes, macrophages and dendritic-like cells were selectively expressed by Arg1^+^ macrophages (Fig. 2e). Given these data, we further examined the transcriptional profile of Arg1^+^ macrophages using pathway analysis. Arg1^+^ macrophages were characterized by the expression of genes involved in prostaglandin synthesis, metabolic regulation (electron transport chain, glycolysis and gluconeogenesis) and cytokine and inflammatory response pathways (Fig. 2f). Transcription factor enrichment predicted that Arg1^+^ macrophage marker genes were regulated by transcription factors involved in inflammatory macrophage development and function (MYB, NCOR, RELA, BRD4, ATF3, SMRT and NRF2) (Fig. 2g)^31–36^. IHC costaining for ARG1 and CD68 (macrophage marker) confirmed the overrepresentation of Arg1^+^ macrophages within the infarct of macHif1aKO mice in comparison with control mice 5 days after I/R (Fig. 2h,i).

**Extended Data Fig. 3 |:**
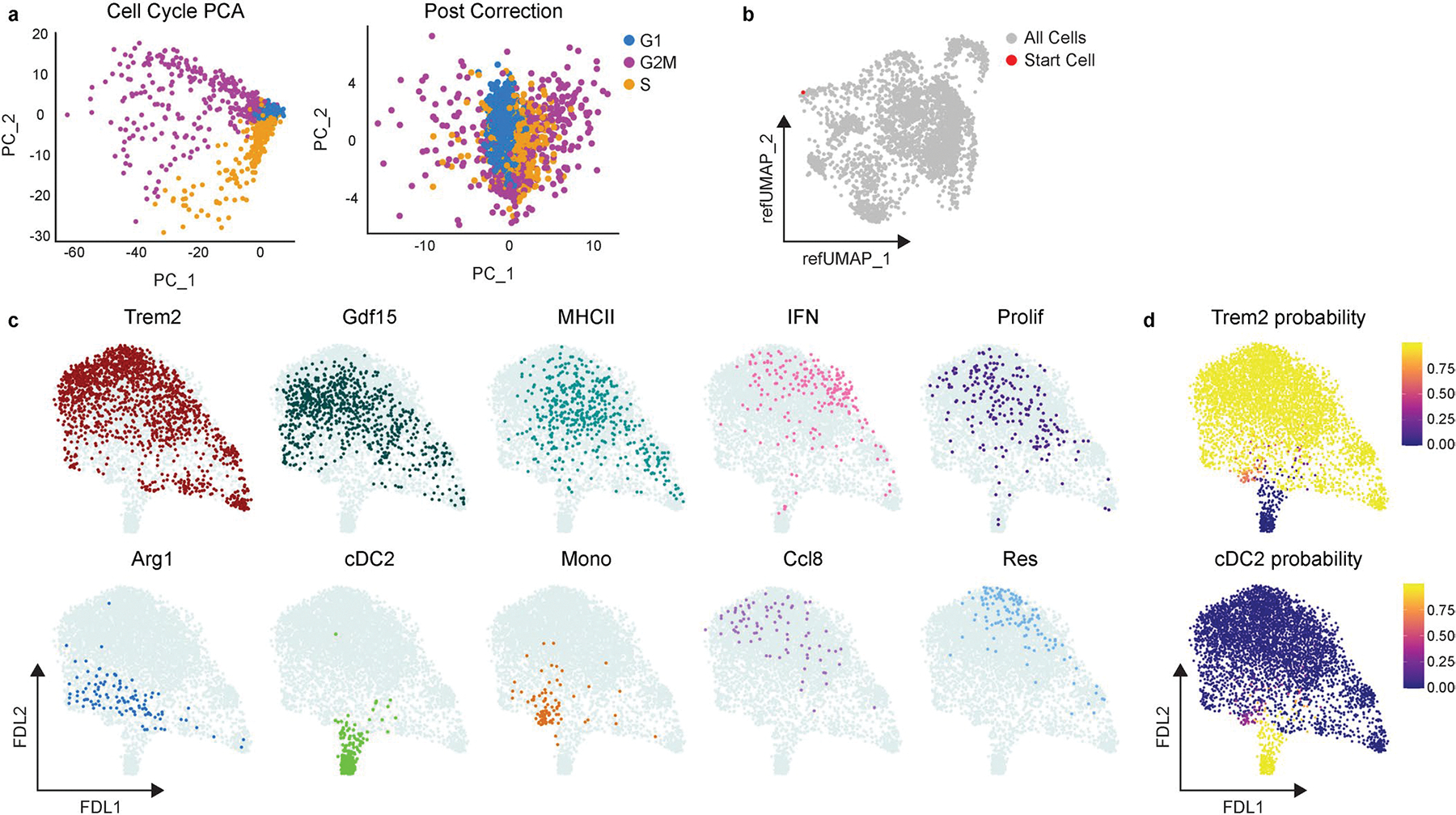
Palantir analysis of Control and macHif1aKO mice 5 days after MI. **a**, PCA plot of cell cycle genes in Control and macHif1aKO scSEQ data 5 days after I/R before (Cell Cycle PCA) and after (Post Correction) regressing out cell cycle scores. **b**, Reference UMAP of Control and macHif1aKO scSEQ data 5 days after I/R highlighting the starting cell used. Starting cell was determined by selecting the Control monocyte with the highest monocyte Z-score (*Plac8*, *Ly6c2*). **c**, Force directed layout (FDL) output of Palantir analysis highlighting each cell subpopulation in Control and macHif1aKO scSEQ data. **d**, Terminal state probability scores from Palantir analysis indicating the likelihood that a cell becomes either Trem2 or cDC2.

To ascertain the differential trajectory of monocytes, macrophages and dendritic-like cells in control and macHif1aKO hearts, we used Palantir trajectory analysis^37^. Monocytes were selected as the least differentiated cell population, and Palantir predicted Trem2^+^ macrophages and cDC2s (type-2 classical dendritic cells) as terminal populations (low entropy and high pseudotime values) (Fig. 2j and Extended Data Fig. 3). As anticipated, monocytes displayed high entropy and low pseudotime values. Arg1^+^ macrophages displayed entropy and pseudotime values in between monocytes and terminal populations, suggesting that they represent intermediates in monocyte differentiation (Fig. 2j,k). These data support the concept that hypoxia sensing in monocytes and/or macrophages influences steps in monocyte fate specification. To validate our findings, we compared our results with public datasets of mouse MI^15,23–25^ (Supplementary Fig. 2a–d). Arg1^+^ macrophages were similar to previously identified intermediate Trem2^hi^Spp1^hi^ macrophages, and we confirmed that the Arg1^+^ signature is upregulated early after injury (Supplementary Fig. 2e–h).

### Origin and spatiotemporal dynamics of Arg1^+^ macrophages

**Fig. 3 |:**
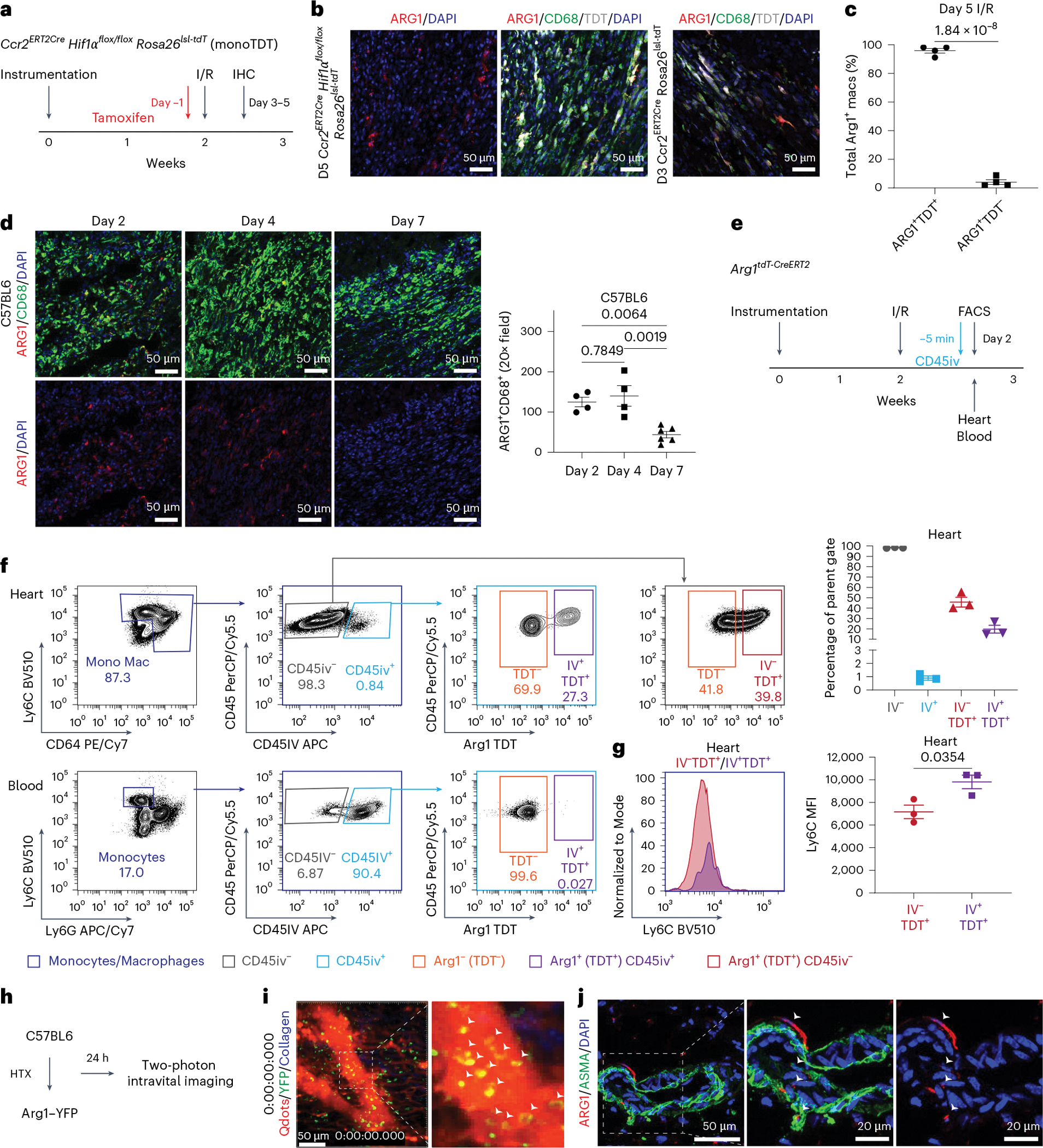
Spatiotemporal dynamics of Arg1^+^ macrophages after ischemic heart injury. **a**, A schematic of the mouse model of MI in monoTDT mice. Tamoxifen (60 mg kg^−1^) was i.p. injected 1 day before I/R. Tissues were collected 3–5 days after I/R for IHC. **b**, Representative 20× confocal image of ARG1 (red), CD68 (green), TDT (white) and DAPI (blue) IHC within the infarcts of monoTDT mice. **c**, Quantification of **b** displayed as the percentage of ARG1^+^CD68^+^ macrophages (macs) that are ARG1^+^TDT^+^ or ARG1^+^TDT^−^. *N* = 4. **d**, Representative 20× confocal images of ARG1 (red), CD68 (green) and DAPI (blue) IHC within the infarct 2, 4 and 7 days after I/R in C57/BL6 mice. Quantification displayed as the total ARG1^+^CD68^+^ cells per 20× field. *N* = 4 versus 4 versus 6 (each data point is an individual mouse). **e**, A schematic of intravascular (IV) staining FACS of *Arg1*^*tdT-CreERT2*^ mice 2 days after I/R. Mice were injected with a CD45 antibody 5 min before tissue collection. **f**, FACS of hearts and blood of *Arg1*^*tdT-CreERT2*^ injected with CD45 antibody 2 days after I/R. The quantification represents the populations of interest. *N* = 3. **g**, A histogram of representative Ly6C staining between CD45iv^−^Arg1^+^ and CD45iv^+^Arg1^+^ cells, and quantification of Ly6C mean fluorescence intensity (MFI) between CD45iv^−^Arg1^+^ and CD45iv^+^Arg1^+^ cells. *N* = 3. **h**, A schematic of intravital two-photon microscopy on coronary veins in *Arg1*^*YFP*^ mice. C57BL6 hearts were syngeneically transplanted (HTX) into *Arg1*^*YFP*^ mice and 24 h later were imaged for 15 min. **i**, Representative two-photon image of syngeneic transplant *Arg1*^*YFP*^ mice. Qdots (red) were injected intravenously before imaging to visualize blood vessels. Collagen is in blue. Intravascular Arg1^+^ cells (green) costained with Qdots are highlighted by arrows. **j**, Confocal image (40×) of IHC costaining of ARG1 (red) and α-SMA^+^ vessels (green) 2 days after I/R highlighted by the white arrow. The image is a maximum orthogonal projection. *P* values were determined using a two tailed *t*-test assuming equal variance or ordinary one-way ANOVA using Tukey’s multiple comparisons test. Data are presented as mean ± s.e.m.

We next validated the above predictions made from scRNAseq analysis. To determine whether Arg1^+^ macrophages are monocyte-derived, we utilized a monocyte fate labeling strategy that labels peripheral monocytes but not cardiac macrophages by administering a single dose of 60 mg kg^−1^ tamoxifen to *Ccr2*^*ERT2Cre*^ *Hif1α*^*flox/flox*^ *Rosa26*^*lsl-tdT*^ (monoTDT) mice. We subjected monoTDT mice to closed-chest I/R and assessed tdTomato (TDT) expression 5 days after I/R (Fig. 3a). Mice were treated with tamoxifen 24 h before I/R. The majority of Arg1^+^ macrophages were TDT^+^ indicating that Arg1^+^ macrophages are monocyte-derived. The same observation was made with *Ccr2*^*ERT2Cre*^ *Rosa26*^*lsl-tdT*^ mice 3 days after I/R (Fig. 3b,c).

We next investigated the spatiotemporal dynamics of Arg1^+^ macrophages. IHC in C57/BL6 hearts subjected to I/R demonstrated Arg1^+^ macrophages are present within the infarct 2 and 4 days after injury, but are largely gone 7 days after injury (Fig. 3d).

**Extended Data Fig. 4 |:**
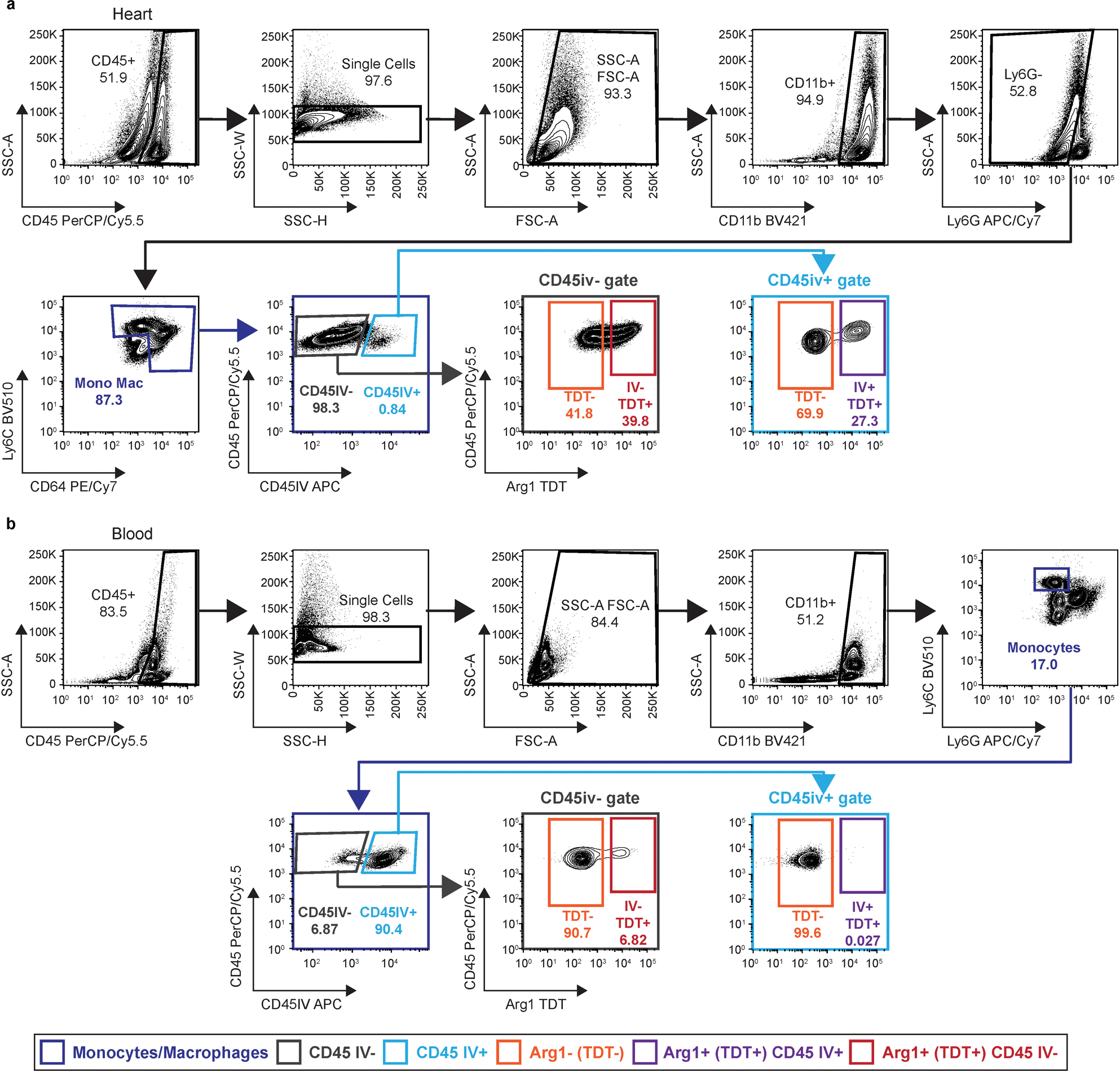
Intravascular staining FACS of *Arg1*^*tdT-CreERT2*^ mice 2 days after MI. **a**, FACS gating strategy for intravascular staining in the heart of *Arg1*^*tdt-CreERT2*^ mice 2 days after I/R. Mice were injected with CD45 antibody 5 minutes prior to tissue collection (CD45IV). Cells were gated for leukocytes (SSC-A vs CD45), single cells (SSC-W vs SSC-H), debris exclusion (SSC-A vs FSC-A), myeloid cells (SSC-A vs CD11b), neutrophil exclusion (SSC-A vs Ly6G) and monocytes/macrophages (Ly6C vs CD64). Intravascular (CD45IV^+^) and extravascular cells (CD45IV^−^) were identified (CD45 vs CD45IV). Arg1^+^ cells were identified via TDT expression in the CD45iv^−^ and CD45iv^+^ gate (CD45 vs Arg1 (TDT)). **b**, Representative FACS plots of the peripheral blood of *Arg1*^*tdt-CreERT2*^ mice 2 days after I/R when performing intravascular staining.

Trajectory analysis predicted that Arg1^+^ macrophages are intermediates in monocyte differentiation. To investigate where Arg1^+^ macrophage specification is initiated, we employed a FACS-based approach to differentially label intravascular versus extravascular leukocytes. *Arg1*^*tdT-CreERT2*^ reporter mice^38^ were analyzed 2 days after MI, a time point where monocytes are robustly recruited to the heart^39^. A fluorescently conjugated CD45 antibody was injected intravascularly into *Arg1*^*tdT-CreERT2*^ reporter mice 5 min before tissue collection, which has previously been shown to differentiate intravascular leukocytes from extravascular leukocytes^40^ (Fig. 3e). We detected Arg1^+^ macrophages within both the extravascular (CD45iv^−^) and intravascular (CD45iv^+^) compartments. Conversely, negligible TDT signal was observed in peripheral monocytes, indicating that Arg1^+^ macrophages are specified within the vasculature of the heart (Fig. 3f and Extended Data Fig. 4). Mean fluorescence intensity of Ly6C staining was higher in Arg1^+^CD45iv^+^ cells compared with Arg1^+^CD45iv^−^ cells, reflecting that intravascular and extravascular Arg1^+^ macrophages are at different stages of monocyte/macrophage differentiation (Fig. 3g). Similar results were found in *Arg1*^*YFP*^ mice (Supplementary Fig. 3). Furthermore, monocyte waterfall analysis of Arg1^+^ macrophages in *Arg1*^*tdT-CreERT2*^ mice 3 days after MI revealed that Arg1^+^ macrophages are at a more intermediate (P2) rather than a more differentiated (P3) stage compared with Arg1^−^ macrophages^41^ (Supplementary Fig. 4).

To verify that Arg1^+^ macrophages are present within the intravascular compartment, we performed intravital two-photon microscopy to visualize Arg1^+^ macrophages in real time. We leveraged a previously established syngeneic heart transplant model of I/R injury in combination with *Arg1* reporter mice^5,42^. C57/BL6 donor hearts were collected, placed on ice for 60 min and then transplanted into *Arg1*^*YFP*^ mice in the cervical position (Fig. 3h). Fluorescently labeled Qdots were injected intravenously and donor hearts were imaged 24 h after transplantation. We observed YFP^+^ cells within the intravascular and extravascular compartments, suggesting that Arg1^+^ macrophage specification is initiated before extravasation into the myocardium (Fig. 3i). IHC confirmed Arg1^+^ cells within α-SMA^+^ vessels in control mice 2 days after I/R (Fig. 3j). Overall, these results reveal that Arg1^+^ macrophages are monocyte-derived and are specified early after monocyte recruitment to the ischemic heart.

### Resident *Hif1α* deletion enriches Arg1^+^ macrophages

**Fig. 4 |:**
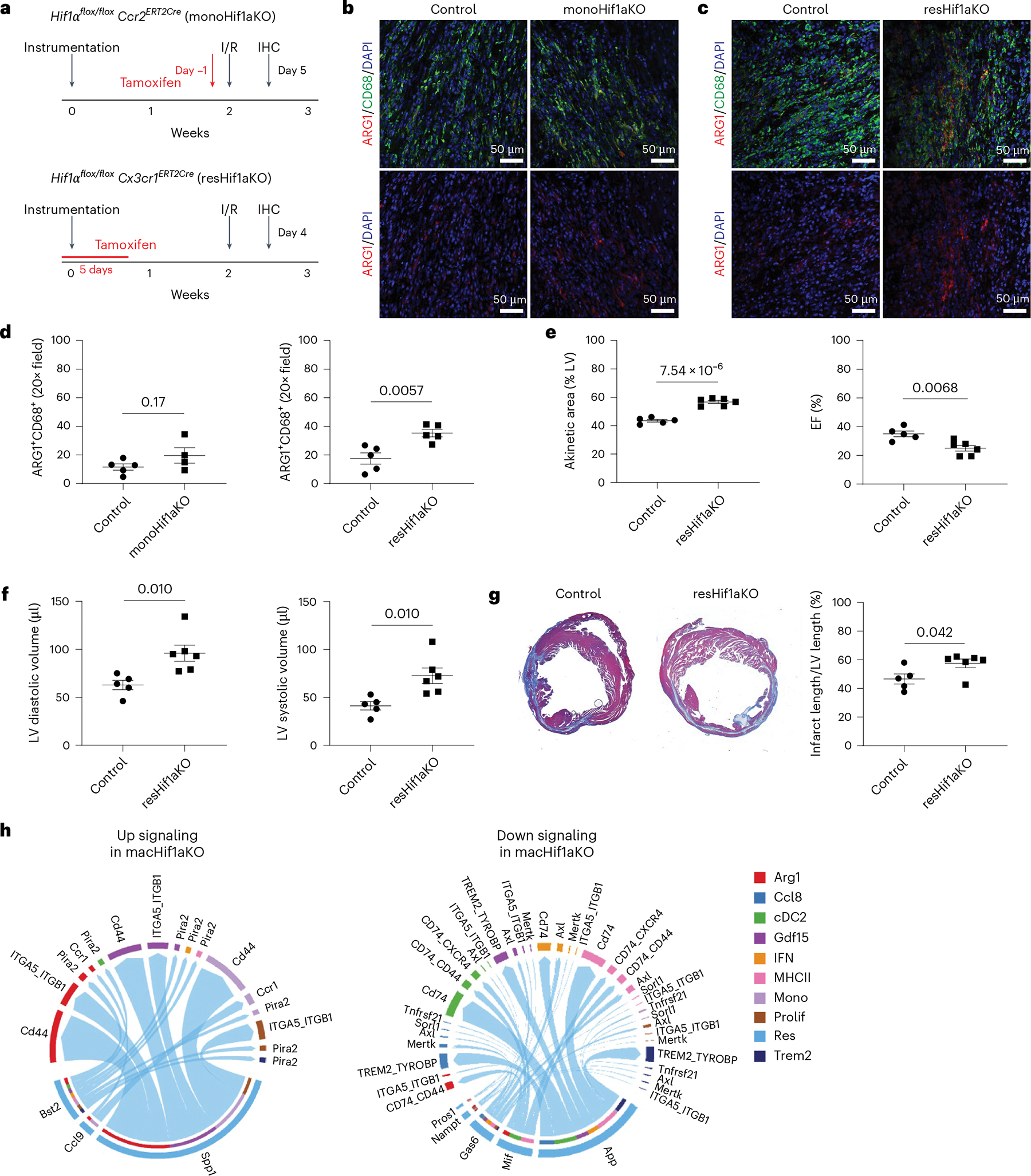
*Hif1α* deletion in cardiac resident macrophages is sufficient to increase the abundance of Arg1^+^ macrophages and accelerate LV remodeling after MI. **a**, Experimental outline of the I/R mouse model of MI in monoHif1aKO and resHif1aKO mice. Closed-chest I/R was performed 2 weeks after instrumentation, and tissues were collected 4–5 days after I/R for analysis. Tamoxifen (60 mg kg^−1^) was i.p. injected 1 day before I/R in monoHif1aKO mice to induce *Hif1α* KO in monocytes and recruited macrophages. Tamoxifen (60 mg kg^−1^) was i.p. injected for 5 days followed by a chase period to induce *Hif1α* KO in resident macrophages. **b**,**c**, Representative 20× confocal images of IHC staining in monoHif1aKO (**b**) and resHif1aKO (**c**) mice. ARG1 is in red, CD68 is in green and DAPI is in blue. **d**, Quantification of IHC in **b** (*N* = 5 versus 4) and **c** (*N* = 5), respectively. Data are displayed as the percentage of ARG1^+^CD68^+^ cells out of total CD68^+^ cells. **e**, Echocardiography quantification 4 weeks after I/R of the akinetic area as a percentage of the LV and EF between control and resHif1aKO mice. **f**, LV diastolic and systolic volume between control and resHif1aKO mice 4 weeks after I/R. **g**, Representative trichrome staining and quantification (displayed as the length of the infarct as a percentage of the total length of the left ventricle) of control and resHif1aKO hearts 4 weeks after I/R. **h**, CellChat analysis of macHif1aKO data 5 days after I/R, represented as a chord diagram of upregulated and downregulated ligand receptor interactions between resident macrophages and other cell types. *P* values were determined using a two tailed *t*-test assuming equal variance. In **e**–**g**, *N* = 5 versus 6. For *N*, each data point is an individual mouse. Data are presented as mean ± s.e.m.

We next investigated whether hypoxia sensing regulates Arg1^+^ macrophage specification in a cell intrinsic or extrinsic manner. To delete *Hif1α* in infiltrating monocytes and their progeny (monoHif1aKO), *Hif1α*^*flox/flox*^ *Ccr2*^*ERT2Cre*^ mice received a single dose of tamoxifen (60 mg kg^−1^, intraperitoneal (i.p.)) 24 h before MI (Fig. 4a). To delete *Hif1α* in resident macrophages (resHif1aKO), we pulsed *Hif1α*^*flox/flox*^ *Cx3cr1*^*ERT2Cre*^ mice with tamoxifen (60 mg kg^−1^ daily for 5 days) immediately after instrumentation to label all monocytes and macrophages. Mice then underwent a chase period of 9 days before MI to allow monocytes to replenish from hematopoietic progenitors, leaving only resident macrophages labeled (Fig. 4a)^5^. IHC staining for CD68 and TDT within the infarct, border and remote zones confirmed that these macrophage subsets occupy distinct locations within the infarcted heart (Supplementary Fig. 5). Quantitative PCR confirmed *Hif1α* loss in monocytes and monocyte-derived macrophages in monoHif1aKO mice (Supplementary Fig. 6).

To delineate whether deleting *Hif1α* in infiltrating monocytes and/or cardiac resident macrophages contributed to expansion of Arg1^+^ macrophages, we quantified Arg1^+^ macrophages by IHC 4–5 days after I/R in monoHif1aKO and resHif1aKO hearts. monoHif1aKO hearts showed no difference compared with control, while resHif1aKO hearts displayed an increase in the abundance of Arg1^+^ macrophages within the infarct (Fig. 4b–d). These data indicate that *Hif1α* in cardiac resident macrophages influence the specification of Arg1^+^ macrophages through a non-cell autonomous mechanism.

**Extended Data Fig. 5 |:**
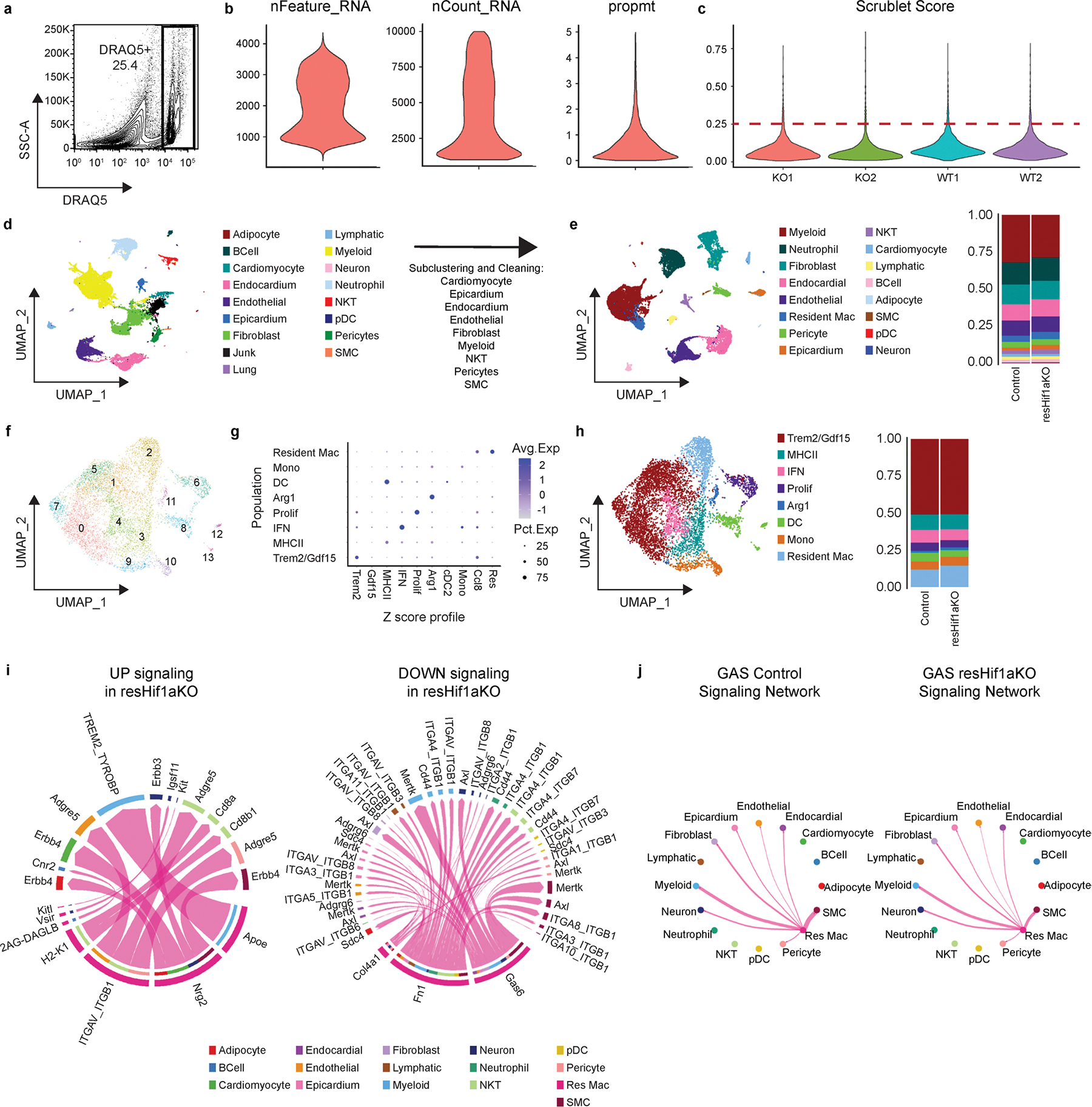
Single nuclei RNA sequencing and clustering of resHif1aKO hearts 3 days after MI. **a**, Representative FACS plot of single nuclei prep from Control and resHif1aKO hearts 3 days after I/R stained with DRAQ5 and used for snRNAseq. **b**, Violin plots representing quality control of snRNAseq data post quality control. Nuclei with greater than 1000 and less than 10000 read counts (nCount_RNA) and proportion of transcripts mapping to mitochondrial genes less than 5 percent (prompt) were used for downstream analysis. **c**, Violin plots of scrublet scores in each snRNAseq library (KO1/KO2 – resHif1aKO, WT1/WT2 – Control). Nuclei with scores less that 0.25 were used for downstream analysis. **d**, UMAP of snRNAseq data after quality control, normalization, clustering, and annotation. **e**, UMAP of cleaned and re-clustered data from d. Cardiomyocyte + Epicardium, Endocardium + Endothelium, Fibroblast, Myeloid, NKT, and Pericyte + SMC populations were subset and individually re-clustered to remove contaminating populations, and then recombined and re-clustered with Adipocyte, BCell, Lymphatic, Neuron, Neutrophil, and pDC populations to generate a final dataset. Stacked bar graph represents proportions of each annotated population per genotype. **f**, UMAP of Myeloid populations after re-clustering. **g**, Z-scores from reference MI scRNAseq data set (monocytes, macrophages, and dendritic-like cells 3, 7, 13, and 28 days after I/R) used to assist with annotating data from f represented as a dot plot. **h**, UMAP and stacked bar graph split by genotype of annotated Myeloid populations. **i**, CellChat analysis of resHif1aKO data 3 days after I/R represented as a chord diagram of upregulated and downregulated ligand receptor interactions between resident macrophages and other cell types. **j**, CellChat chord diagram representing the GAS signaling network in Control and resHif1aKO mice from resident macrophages to other cell types.

To gain insights into the paracrine signaling from resident macrophages to differentiating macrophages, we analyzed our macHif1aKO scRNAseq data with CellChat receptor–ligand analysis. Resident macrophages differentially signaled to other subsets of macrophages between conditions, including downregulated Gas6 signaling in macHif1aKO mice (Fig. 4h). However, resident macrophages should also influence other cardiac cell types^43^. We performed single-nuclei RNA sequencing (snRNAseq) on control and resHif1aKO mouse hearts 3 days after I/R to assess all cell types in the heart at a time point when Arg1^+^ macrophages are beginning to differentiate (Extended Data Fig. 5a–e and Supplementary Tables 2 and 3). Arg1^+^ macrophages were overrepresented in resHif1aKO nuclei, but their total fraction was lower than anticipated, reflecting the lower sensitivity of snRNAseq data (Extended Data Fig. 5f–h). CellChat predicted differential signaling from resident macrophages in the snRNAseq data, including downregulated Gas6 signaling in resHif1aKO mice from resident macrophages to several cardiac cell types (Extended Data Fig. 5i,j). Thus, the non-cell autonomous impact of *Hif1α* deletion is probably more complex than direct signaling from resident cardiac macrophages to differentiating monocytes.

We next examined whether deletion of *Hif1α* in resident macrophages contributes to LV remodeling. Echocardiography and trichrome staining 4 weeks post-I/R revealed that resHif1aKO mice had increased akinetic LV area, reduced LV EF, increased LV diastolic and systolic volume and increased infarct size (Fig. 4e–g). There was no significant difference in cardiomyocyte hypertrophy (Supplementary Fig. 7). Deletion of *Hif1α* in resident cardiac macrophages did not increase resident cardiac macrophage cell death (Supplementary Fig. 8). These findings indicate that deletion of *Hif1α* in resident cardiac macrophages is sufficient to recapitulate many of the phenotypes observed in mice lacking *Hif1α* in all monocytes/macrophages and that hypoxia sensing in cardiac resident macrophages regulates the specification of infiltrating monocytes.

### Lineage tracing of Arg1^+^ macrophages during MI

**Extended Data Fig. 6 |:**
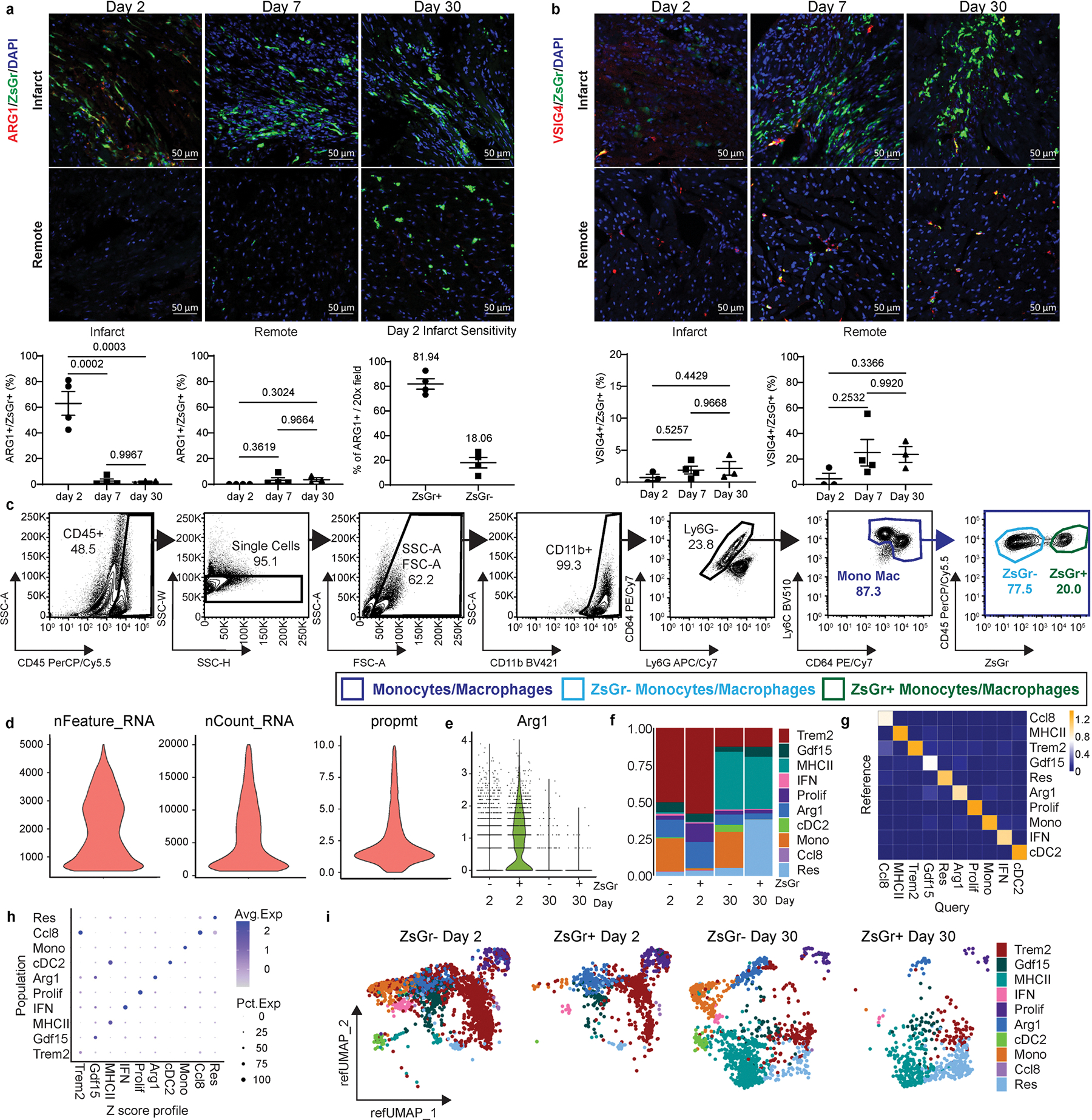
Validation, single cell RNA sequencing, and reference mapping of Arg1ZsGr mice after MI. **a**, Representative 20x confocal images of ARG1 IHC in the infarct and remote zone of Arg1ZsGr mice 2, 7, 30 days after I/R. Quantification of IHC is displayed as the percentage of ZsGr^+^ cells that are ARG1^+^ZsGr^+^. N = 4 vs 4 vs 3. Sensitivity of Arg1ZsGr mice 2 days after I/R within the infarct, quantified as the percentage of ARG1^+^ cells that are ZsGr^+^ or ZsGr^−^. N = 4 vs 4. **b**, Representative 20x confocal images of VSIG4 IHC in the infarct and remote zone of Arg1ZsGr mice 2, 7, 30 days after I/R. Quantification of IHC is displayed as the percentage of ZsGr^+^ cells that are VSIG4^+^ZsGr^+^. N = 3 vs 4 vs 3. **c**, FACS gating strategy for isolation of ZsGr^−^ and ZsGr^+^ monocytes and macrophages from Arg1ZsGr mice 2 and 30 days after I/R. **d**, Violin plots representing scRNAseq data post quality control. Cells with greater than 500 and less than 5000 genes (nFeature_RNA), read counts less than 20,000 (nCount_RNA), and proportion of transcripts mapping to mitochondrial genes less than 10 percent (prompt) were used. **e**, *Arg1* expression in the ZsGr^−^ and ZsGr^+^ libraries at day 2 and 30. **f**, Stacked bar graph representing the proportion of each cell population in the data split between ZsGr^−^ and ZsGr^+^ cells 2 and 30 days after I/R. **g**, Heat map of mapping scores for each cell population in the Query (Arg1ZsGr) plotted against the Reference MI data set. **h**, Z-score profiles from the reference data set (Extended Data Fig. 2f) compared to each subpopulation in the Arg1ZsGr data set represented as a dot-plot. **i**, Reference UMAP plots split between ZsGr^−^ and ZsGr^+^ libraries 2 and 30 days after I/R after mapping to reference MI data set. The reference MI scRNAseq data set was the same as the one displayed in Extended Data Fig. 2. P-values are determined using the ordinary one-way ANOVA using Tukey’s multiple comparisons test. For N, each data point is an individual mouse. Data are presented as mean ± SEM.

**Fig. 5 |:**
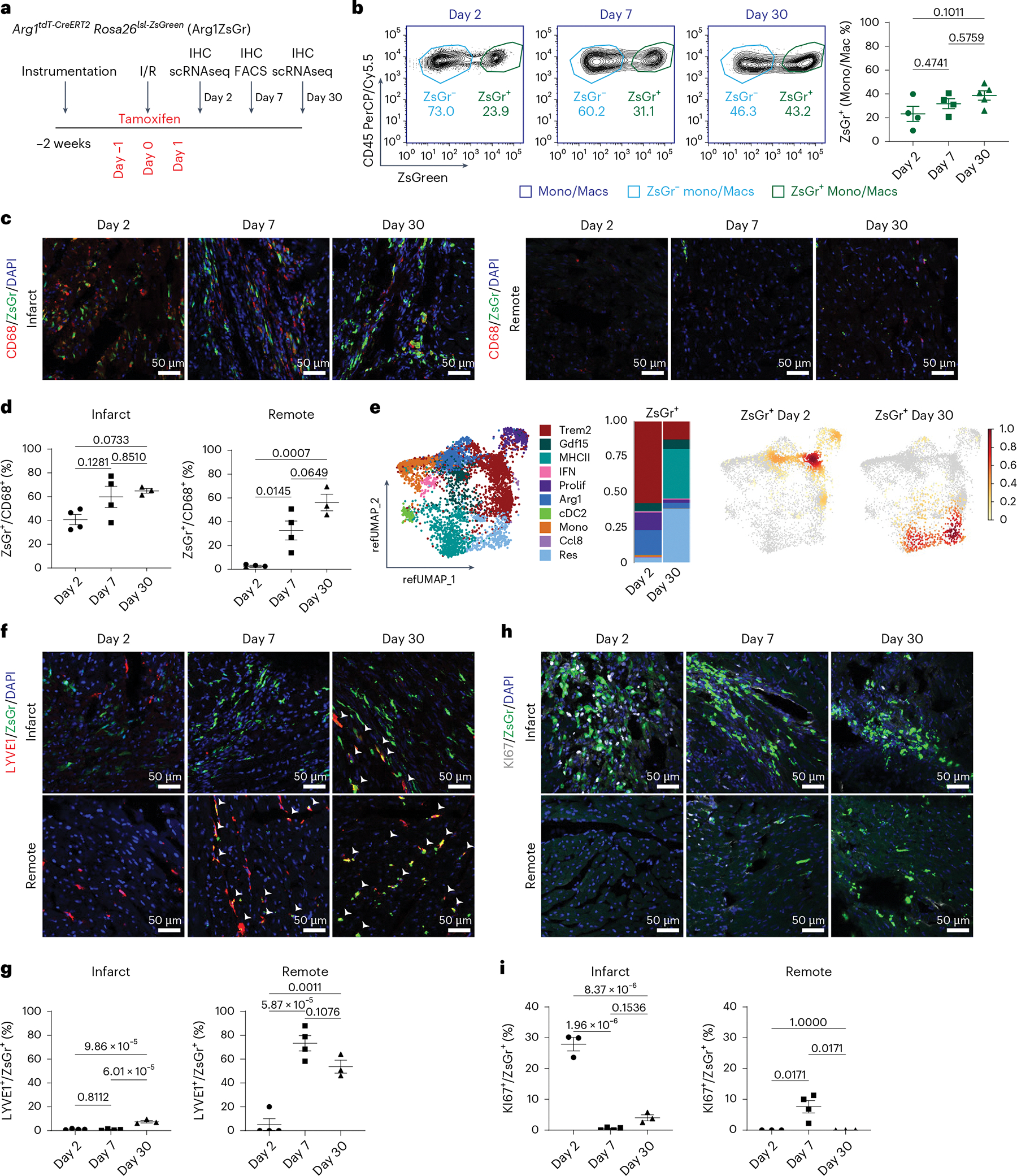
The Arg1 macrophage differentiation trajectory is maintained through MI and differentiates into various macrophage subtypes. **a**, Experimental outline of the I/R mouse model of MI in Arg1ZsGr mice used to lineage trace Arg1 macrophages. Closed-chest I/R was performed 2 weeks after instrumentation, and tissues were collected 2, 7 and 30 days after I/R for FACS and IHC. ZsGr^−^ and ZsGr^+^ monocytes and macrophages were collected 2 and 30 days after I/R for scRNAseq. Tamoxifen (60 mg kg^−1^) was i.p. injected at −1, 0 and +1 days relative to the time of injury. **b**, Representative FACS and quantification of ZsGr^−^ and ZsGr^+^ macrophages in Arg1ZsGr mice 2, 7 and 30 days after I/R. A monocyte/macrophage (Mono/Mac) back gate was used. *N* = 4 versus 4 versus 5. **c**, Representative 20× confocal images of Arg1^+^ macrophage lineage tracing IHC in the infarct and remote zones of Arg1ZsGr mice 2, 7 and 30 days after I/R. CD68 staining is in red, ZsGr is in green and DAPI is in blue. **d**, Quantification of IHC in **c**. Data are displayed as the percentage of ZsGr^+^CD68^+^ cells out of total CD68^+^ cells per 20× field. *N* = 4 versus 4 versus 3. **e**, Annotated reference UMAP, Gaussian kernel density estimate plots and stacked bar graph of ZsGr^+^ scRNAseq data 2 and 30 days after I/R in Arg1ZsGr mice. Data were mapped to a reference MI dataset of monocytes, macrophages and dendritic-like cells 3, 7, 13 and 28 days after I/R. **f**, Representative 20× confocal images of ZsGr^+^ resident macrophages in the infarct and remote zones of Arg1ZsGr mice 2, 7 and 30 days after I/R. The resident macrophage marker LYVE1 is in red, ZsGr is in green and DAPI is in blue. Examples of LYVE1^+^ZsGr^+^ cells are highlighted by white arrows. **g**, Quantification of IHC in **f**. Data are displayed as the percentage of ZsGr+ cells that are LYVE1^+^ZsGr^+^ per 20× field. *N* = 4 versus 4 versus 3. **h**, Representative 20× confocal images of ZsGr^+^ macrophages that are proliferating. The proliferation marker KI67 is in white, ZsGr is in green and DAPI is in blue. **i**, Quantification of **h**. *N* = 3 versus 4 versus 3. *P* values were determined using ordinary one-way ANOVA using Tukey’s multiple comparisons test. For *N*, each data point is an individual mouse. Data are presented as mean ± s.e.m.

Trajectory analysis predicted that Arg1^+^ macrophages are intermediates that give rise to additional macrophage subsets. To validate this, we bred *Arg1*^*tdT-CreERT2*^ *Rosa26*^*lsl-ZsGreen*^ mice^38,44^ (Arg1ZsGr) to track Arg1^+^ cells and their progeny after MI. As Arg1^+^ macrophages are specified early after MI, we administered tamoxifen (60 mg kg^−1^ i.p.) to Arg1ZsGr mice before and immediately after I/R (days −1, 0 and +1) to label and track their fates. Tissues were collected 2, 7 and 30 days after I/R for IHC and FACS. ZsGr^−^ and ZsGr^+^ monocytes and macrophages were collected 2 and 30 days after I/R for scRNAseq (Fig. 5a). ARG1 colocalized with ZsGr^+^ cells specifically within the infarct 2 days after I/R at a sensitivity of 81.94%. At later time points, no ARG1 expression was observed in ZsGr^+^ cells (Extended Data Fig. 6a). ZsGr^+^ monocytes and macrophages persisted 2, 7 and 30 days after I/R via FACS, indicating that cells derived from Arg1^+^ macrophages are maintained throughout MI (Fig. 5b). This was corroborated by IHC costaining of CD68 with ZsGr^+^ cells within the infarct (Fig. 5c,d). ZsGr^+^CD68^+^ cells were also found in the remote zone 7 and 30 days after MI (Fig. 5c,d). ZsGr^+^ cells were not detected in the peripheral blood of Arg1ZsGr mice 2, 7 or 30 days after MI (Supplementary Fig. 9).

scRNAseq data of ZsGr^−^ and ZsGr^+^ monocytes and macrophages were mapped to our reference MI dataset to maintain consistent annotations across experiments (Extended Data Fig. 6c–I and Supplementary Table 4). *Arg1* expression was highest in ZsGr^+^ cells at day 2, consistent with the premise that ZsGr labels Arg1^+^ macrophages (Extended Data Fig. 6e). Composition analysis showed that 2 days after MI, ZsGr^+^ cells consisted primarily of Arg1^+^, proliferating, Trem2^+^ and Gdf15^+^ macrophages. At 30 days after MI, ZsGr^+^ cells mainly comprised MHC-II^hi^ and resident-like macrophages, with some Trem2^+^ and Gdf15^+^ macrophages (Fig. 5e). The contribution of Arg1^+^ macrophages to a resident-like population was validated by LYVE1 (resident macrophage marker) IHC. LYVE1^+^ZsGr^+^ cells were detected in the infarct 30 days after MI and in the remote zone 7 and 30 days after MI (Fig. 5f,g). VSIG4, another resident macrophage marker, also costained with ZsGr^+^ cells (Extended Data Fig. 6b). Additionally, ZsGr^+^ cells included proliferating Ki67^+^ macrophages, suggesting that cells derived from Arg1^+^ macrophages are maintained in part by proliferation (Fig. 5e,h,i). These findings indicate that Arg1^+^ macrophages specified early after MI differentiate into multiple downstream macrophage subsets including a population that resembles cardiac resident macrophages.

### Arg1^+^ macrophages differentiate along a distinct trajectory

**Extended Data Fig. 7 |:**
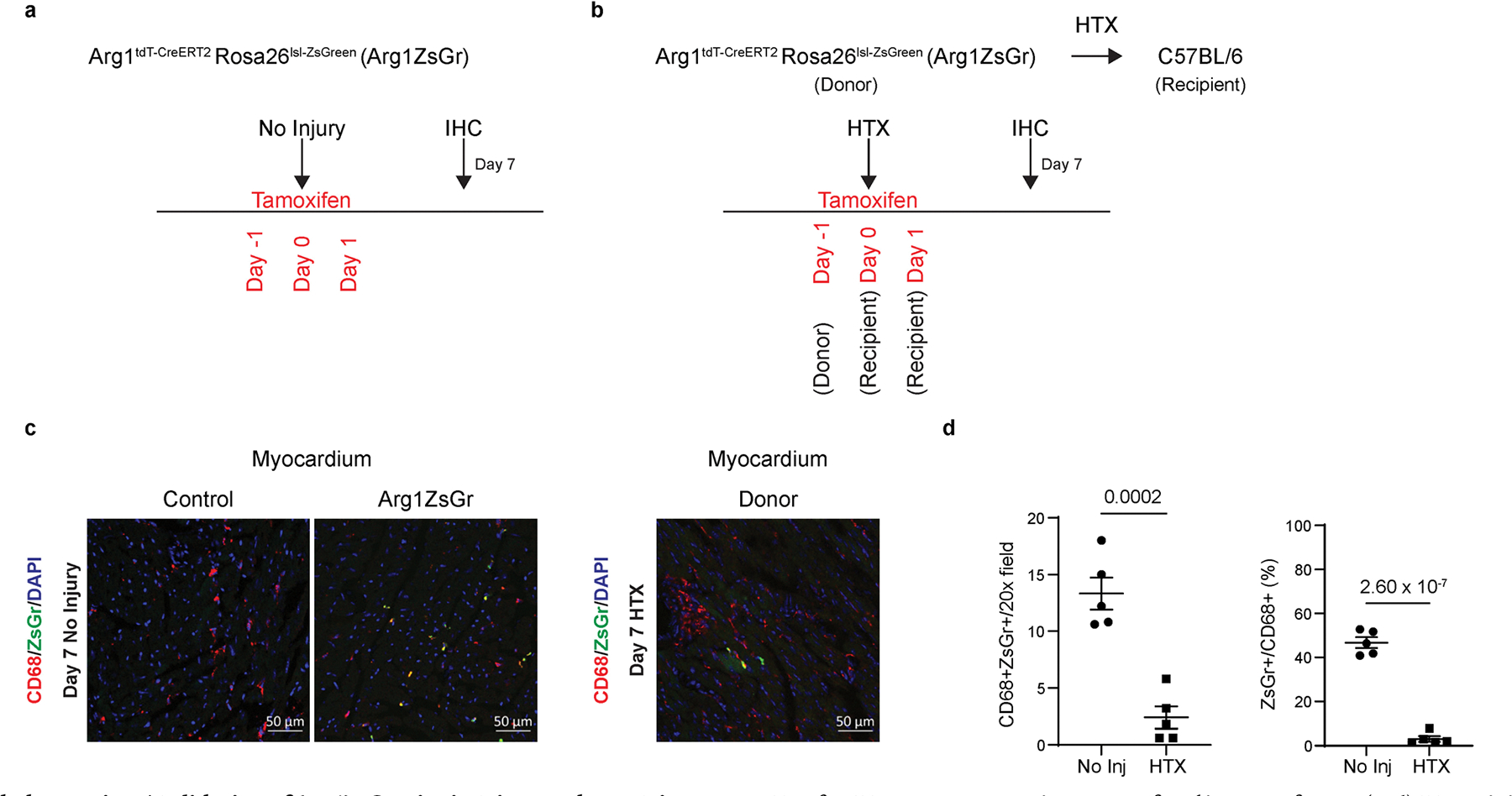
Validation of Arg1ZsGr mice in Injury and Non-Injury conditions. **a**, Schematic of no injury in Arg1ZsGr mice. 60 mg/kg of tamoxifen was injected i.p. and tissues were collected for IHC Day 7 after not inducing injury. **b**, Schematic of Arg1ZsGr donor heart syngeneic transplant (HTX) into C57BL/6 mice. Arg1ZsGr donor mice were given 60 mg/kg tamoxifen via gavage 1 day prior to transplant. C57BL/6 recipient mice were given 60 mg/kg tamoxifen via gavage 0 and +1 days relative to HTX. Donor hearts were collected 7 days after HTX for IHC. **c**, Representative 20x confocal images of CD68 (red) IHC staining in Control, uninjured Arg1ZsGr, and donor Arg1ZsGr myocardium. Endogenous ZsGr is in green, and DAPI is in blue. **d**, Quantification of IHC in c. Data is displayed as total CD68^+^ZsGr^+^ cells per 20x field, and the percentage of CD68^+^ cells that are CD68^+^ZsGr^+^. N = 5 (each data point is an individual mouse). P-values are determined using a two tailed t-test assuming equal variance. Data are presented as mean ± SEM.

The finding that ZsGr^+^CD68^+^ cells were present in the infarct and remote zones after MI is consistent with the premise that Arg1^+^ macrophages and their progeny constitute a previously unappreciated trajectory of macrophage differentiation. Alternatively, this observation could be explained by transient expression of *Arg1* in resident macrophages following MI. To examine this possibility, we transplanted Arg1ZsGr donor hearts into syngeneic recipients and administered tamoxifen (60 mg kg^−1^ i.p.) before and immediately after transplant (days −1, 0 and +1) to label donor resident macrophages that might express *Arg1*. We detected few CD68^+^ZsGr^+^ cells 7 days following transplantation within the myocardium, indicating that cardiac resident macrophages do not express *Arg1* in response to I/R. It is important to note that uninjured hearts contained CD68^+^ZsGr^+^ cells 7 days after tamoxifen administration (Extended Data Fig. 7).

**Extended Data Fig. 8 |:**
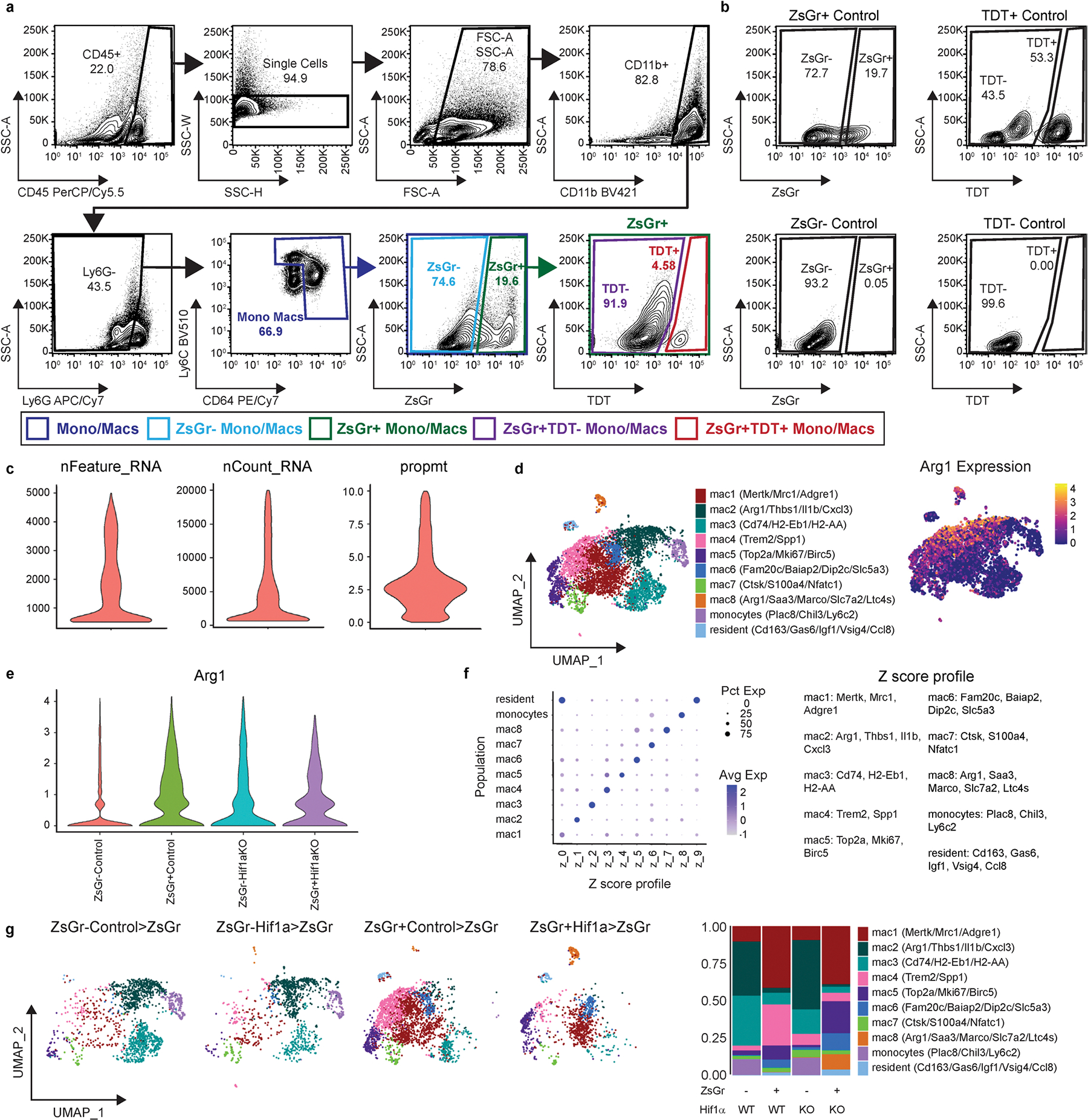
Single cell RNA sequencing of ZsGr^−^ and ZsGr^+^ cells in Control>ZsGr and Hif1a>ZsGr syngeneic heart transplant mice. **a**, FACS gating strategy for isolation of ZsGr^−^ and ZsGr^+^ monocytes and macrophages from Control>ZsGr and Hif1a>ZsGr mice 5 days after syngeneic heart transplant (HTX) for use in scRNAseq. **b**, ZsGr positive and negative controls and TDT positive and negative controls which were used to determine gating for a. **c**, Violin plots representing scRNAseq data post quality control. Cells with greater than 500 and less than 5000 genes (nFeature_RNA), read counts greater than 500 and less than 20,000 (nCount_RNA), and proportion of transcripts mapping to mitochondrial genes less than 10 percent (prompt) were used for downstream analysis. **d**, Annotated UMAP of ZsGr^−^ and ZsGr^+^ cells in Control>ZsGr and Hif1a>Zsgr conditions 5 days after HTX. Arg1 expression plotted on a UMAP projection. **e**, Violin plot of *Arg1* expression split between ZsGr^−^ and ZsGr^+^ cells in Control>ZsGr and Hifa1>ZsGr mice. **f**, Z-score profiles from of each subpopulation in the Control>ZsGr and Hifa1>ZsGr data set represented as a dot-plot. Z-score profile genes for each subpopulation are listed. **g**, Annotated UMAP of scRNAseq data 5 days after HTX split between ZsGr^−^ and ZsGr^+^ cells in Control>ZsGr and Hifa1>ZsGr mice, and stacked bar graph representing the proportion of each cell population in the data set split between conditions.

**Fig. 6 |:**
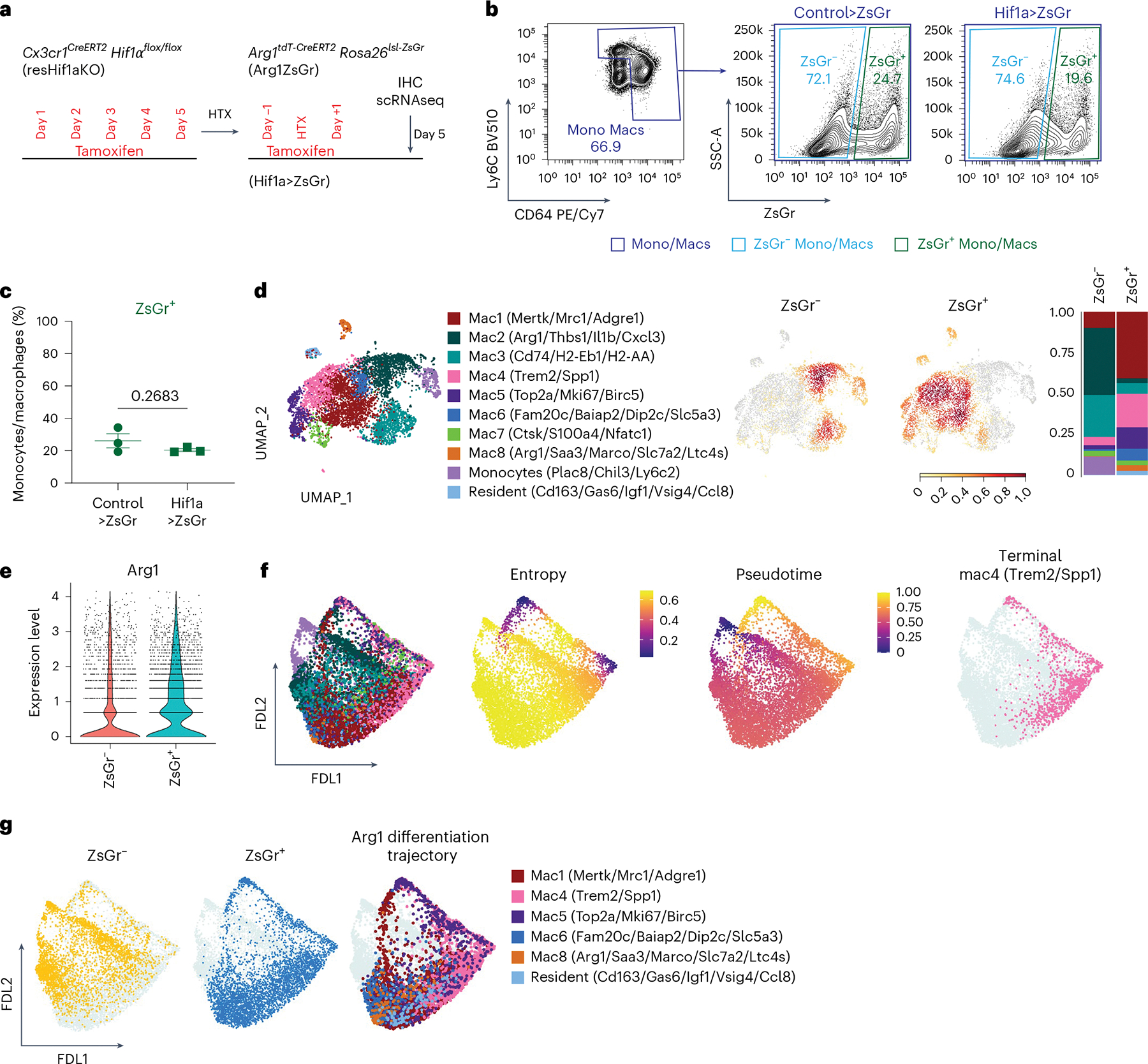
The Arg1 macrophage differentiation trajectory represents a distinct monocyte to macrophage differentiation trajectory during ischemic heart injury. **a**, Experimental outline of the syngeneic heart transplant model used to lineage trace Arg1^+^ macrophages when the donor heart is either a control or resident macrophage *Hif1α* KO (resHif1aKO) heart. Tamoxifen (60 mg kg^−1^) was administered via gavage for 5 days in control and resHif1aKO mice and then transplanted into Arg1ZsGr mice (control>ZsGr or Hif1a>ZsGr). Tamoxifen (60 mg kg^−1^) was administered via gavage to recipient Arg1ZsGr mice at −1, 0 and +1 days relative to the time of transplant. Tissues were collected 5 days after transplant for IHC, FACS and scRNAseq. ZsGr^−^ and ZsGr^+^ monocytes and macrophages were collected for scRNAseq. **b**, Representative FACS of ZsGr^−^ and ZsGr^+^ monocytes and macrophages in control>ZsGr and Hif1a>ZsGr mice. **c**, Quantification of the percentage of ZsGr^+^ monocytes and macrophages in control>ZsGr and Hif1a>ZsGr mice. *N* = 3 (each data point is an individual mouse). Data are presented as mean ± s.e.m. **d**, Annotated UMAP, Gaussian kernel density estimate plots and stacked bar graph of scRNAseq data in control>ZsGr and Hif1a>ZsGr mice, split by ZsGr^−^ and ZsGr^+^ cells. **e**, A violin plot of *Arg1* expression in control>ZsGr and Hif1a>ZsGr mice, split by ZsGr^−^ and ZsGr^+^ cells. **f**, FDL representation of Palantir analysis displaying all the cells as annotated in **d**, entropy and pseudotime scores, and the predicted terminal state mac4 (Trem2/Spp1). **g**, FDL layout of Palantir analysis split by ZsGr^−^ and ZsGr^+^ cells, and cells overrepresented in ZsGr^+^ libraries (Arg1 differentiation trajectory; mac1, mac4, mac5, mac6, mac8 and resident macrophages). *P* values were determined using a two tailed *t*-test assuming equal variance.

To avoid possibly labeling cardiac resident macrophages, we repeated our Arg1^+^ lineage tracing studies using the syngeneic heart transplant model of ischemic heart injury^45–47^. Donor C57BL6 hearts were transplanted into Arg1ZsGr recipients to ensure that ZsGr^+^ cells would be derived from monocytes. This transplant model also provided the opportunity to investigate the impact of *Hif1α* deletion in resident macrophages on Arg1^+^ macrophage differentiation by transplanting either control or resHif1aKO donor hearts into Arg1ZsGr recipients. Tamoxifen was administered to control and resHif1aKO donor mice (60 mg kg^−1^ gavage for 5 days) to delete *Hif1α* in donor (cardiac resident) macrophages. Donor hearts were then placed on ice for 60 min (ischemia) and transplanted into recipient Arg1ZsGr mice (control>ZsGr and Hif1a>ZsGr). Tamoxifen (60 mg kg^−1^) was administered before and immediately after transplant (days −1, 0 and +1) to label and track recipient monocyte-derived Arg1^+^ macrophages. Donor hearts were collected 5 days after transplant for IHC, FACS and scRNAseq (Fig. 6a). Consistent with the existence of a Arg1^+^ macrophage differentiation trajectory, FACS revealed that control and resHif1aKO transplanted hearts contained both ZsGr^−^ and ZsGr^+^ macrophages (Fig. 6b,c and Extended Data Fig. 8a,b).

We then generated scRNAseq libraries from ZsGr^−^ and ZsGr^+^ monocytes and macrophages in control>ZsGr and Hif1a>ZsGr mice. Clustering identified ten transcriptionally distinct subsets of monocytes and macrophages (Extended Data Fig. 8c–g and Supplementary Table 5). We first compared ZsGr^−^ with ZsGr^+^ cells by composition analysis and found that they were transcriptionally distinct. ZsGr^+^ cells displayed a greater abundance of mac1 (*Mertk*, *Mrc1* and *Adgre1*), mac4 (*Trem2* and *Spp1*), mac5 (*Top2a*, *Mki67* and *Birc5*), mac6 (*Fam20c*, *Baiap2*, *Dip2c* and *Slc5a3*), mac8 (*Arg1*, *Saa3*, *Marco*, *Slc6a2* and *Ltc4s*) and resident-like macrophage (*Cd163*, *Gas6*, *Igf1*, *Vsig4* and *Ccl8*) subsets. These data indicate that macrophages following the Arg1^+^ differentiation trajectory differ from other monocyte-derived macrophages (Fig. 6d). These data also recapitulated several key findings observed in our Arg1^+^ lineage tracing studies performed in MI: the presence of Trem2^+^, proliferating and resident-like macrophages. Consistent with the expectation that ZsGr would label Arg1^+^ macrophages, we observed higher *Arg1* expression in ZsGr^+^ cells (Fig. 6e).

**Extended Data Fig. 9 |:**
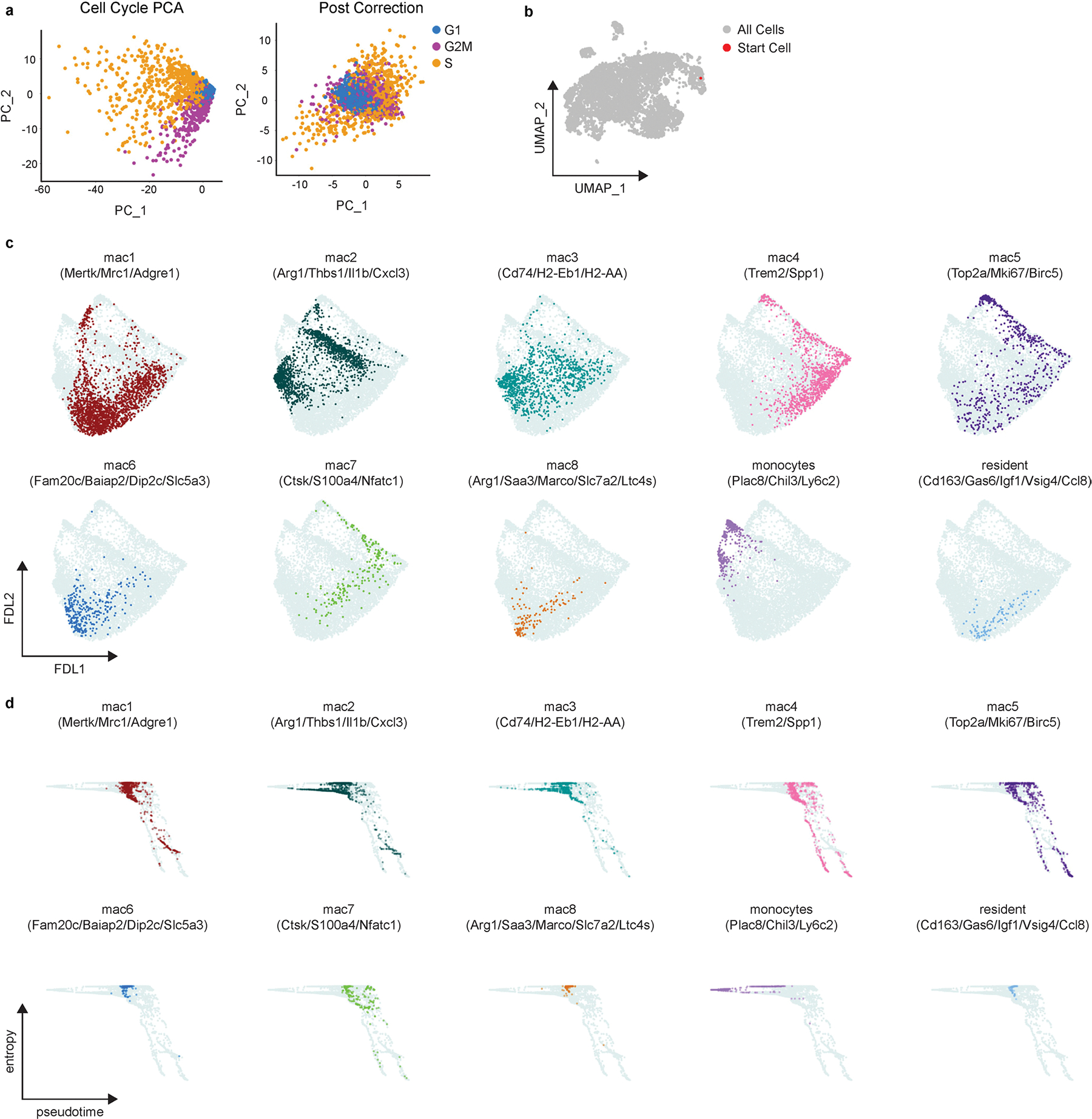
Palantir analysis of ZsGr^−^ and ZsGr^+^ cells in Control>ZsGr and Hif1a>ZsGr syngeneic heart transplant mice. **a**, PCA plot of cell cycle genes in ZsGr^−^ and ZsGr^+^ cells in Control>ZsGr and Hif1a>ZsGr 5 days after syngeneic heart transplant (HTX) before (Cell Cycle PCA) and after (Post Correction) regressing out cell cycle scores. **b**, UMAP projection of scRNAseq data 5 days after HTX highlighting the starting cell used. Starting cell was determined by selecting the monocyte with the highest monocyte Z-score (*Plac8*, *Ly6c2*). **c**, Force directed layout (FDL) output of Palantir analysis highlighting each cell subpopulation in the ZsGr^−^ and ZsGr^+^ cells in Control>ZsGr and Hif1a>ZsGr 5 days after HTX. **d**, Plots of entropy vs pseudotime scores from Palantir analysis for every cell subpopulation in the scRNAseq data set.

Palantir trajectory analysis^37^ was performed on ZsGr^−^ and ZsGr^+^ cells, with monocytes selected as the starting cell type (Extended Data Fig. 9). Palantir suggested that Arg1^+^ macrophages are an intermediate macrophage state and that the Arg1^+^ differentiation trajectory diverged from other monocytes and macrophages present within the transplanted heart. This analysis also predicted that mac4 (*Trem2* and *Spp1*) represented a terminally differentiated state (Fig. 6f,g). Collectively, these findings indicate that Arg1^+^ macrophages are derived from monocytes that infiltrate the heart after ischemic injury and subsequently differentiate into several subsets including Trem2^+^, proliferating and resident-like macrophages.

### Resident macrophages govern Arg1^+^ macrophage differentiation

**Fig. 7 |:**
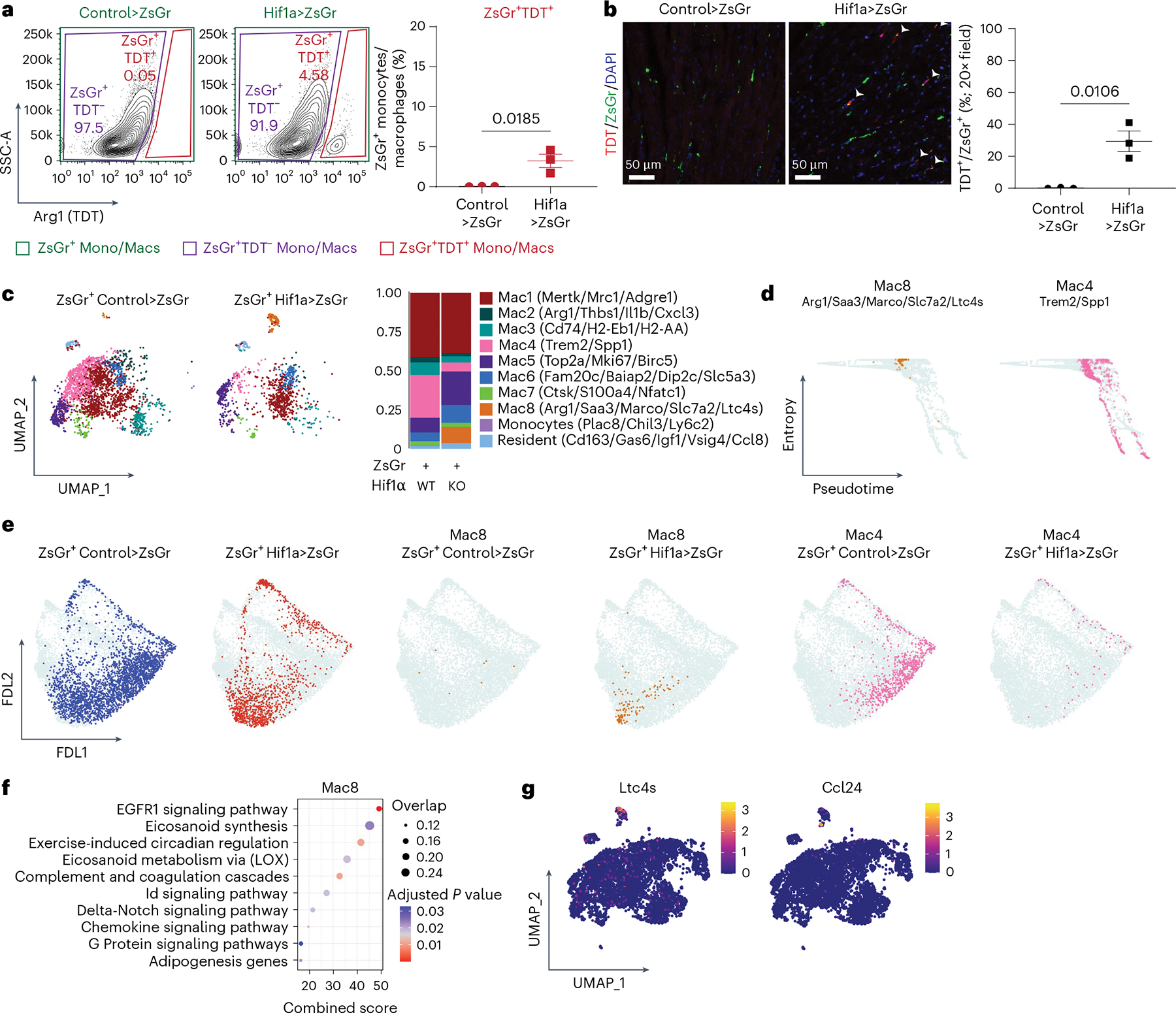
*Hif1α* activity in resident cardiac macrophages regulates progression through the Arg1 differentiation trajectory. **a**, Representative FACS of ZsGr^+^tdT^+^ monocytes and macrophages in control>ZsGr and Hif1a>ZsGr mice. ZsGr^+^ monocytes and macrophages were used as a back gate. Quantification is displayed as the percentage of ZsGr^+^ monocytes and macrophages that are TDT^+^. **b**, Representative 20× confocal images of endogenous ZsGr (green) and TDT (red) IHC in control>ZsGr and Hif1a>ZsGr mice. DAPI is in blue. Costaining of ZsGr with TDT is highlighted by white arrows. Quantification is displayed as the percentage of ZsGr^+^ cells that are ZsGr^+^TDT^+^. In **a** and **b**, *N* = 3 (each data point is an individual mouse). **c**, Annotated UMAP and stacked bar graph of ZsGr^+^ cells split by control>ZsGr (ZsGr^+^, *Hif1α* WT (wildtype) and Hif1a>ZsGr (ZsGr^+^, *Hif1α* KO) conditions. **d**, Plots of entropy versus pseudotime scores from Palantir analysis for Arg1^+^ (mac8) and Trem2^+^ (mac4) macrophages. **e**, FDL layout of Palantir analysis of ZsGr^+^ cells split by control>ZsGr (blue) and Hif1a>ZsGr (red) conditions. Populations of interest (mac8, orange and mac4, pink) plotted on a FDL layout split by control>ZsGr and Hif1a>ZsGr conditions. **f**, A dot plot of the top ten upregulated pathways (Enrichr Wikipathways 2019 Mouse) in mac8 (Arg1, Saa3, Marco, Slc7a2 and Ltc4s) cells ordered by the combined score. **g**, Gene expression of *Ltc4s* (eicosanoid synthesis term) and *Ccl24* (chemokine signaling pathway term) plotted on a UMAP. *P* values were determined using a two tailed *t*-test assuming equal variance. Data are presented as mean ± s.e.m.

Our studies showed that *Hif1α* deletion in resident macrophages increased the abundance of monocyte-derived Arg1^+^ macrophages. To ascertain how Arg1^+^ macrophages accumulate in this setting, we compared ZsGr^+^ macrophages from control and resHif1aKO donor hearts that were transplanted into Arg1ZsGr mice (control>ZsGr and Hif1a>ZsGr) (Fig. 6a). FACS and IHC analysis of ZsGr^+^ monocytes and macrophages revealed that Hif1a>ZsGr had higher amounts of TDT^+^ cells 5 days after transplant (Fig. 7a,b and Extended Data Fig. 8a,b). These data indicate increased abundance of ZsGr^+^ cells expressing *Arg1* in resHif1aKO donor hearts, consistent with our MI data showing accumulation of Arg1^+^ macrophages when *Hif1α* is deleted from resident macrophages.

To determine whether progression through the Arg1^+^ differentiation trajectory is interrupted in resHif1aKO donor hearts, we next compared the composition of ZsGr^+^ cells between control and resHif1aKO donor hearts. resHif1aKO donor hearts displayed lower proportions of Trem2^+^ macrophages (mac4) and greater proportions of proliferating (mac5) and Arg1^+^ macrophages (mac8) (Fig. 7c). Palantir trajectory analysis predicted that Arg1^+^ macrophages (mac8) represent intermediates (high entropy, low pseudotime) and Trem2^+^ macrophages (mac4) represent a terminal state (low entropy, high pseudotime) (Fig. 7d). Visualization of ZsGr^+^ cells within control and resHif1aKO donor hearts in trajectory space signaled a bias toward Trem2^+^ macrophages and Arg1^+^ macrophages, respectively (Fig. 7e). In addition, Arg1^+^ macrophages displayed inflammatory gene signatures such as eicosanoid synthesis and chemokine signaling (Fig. 7f,g). Collectively, these findings demonstrate that Hif1α signaling in resident cardiac macrophages regulates the differentiation of Arg1^+^ macrophages. *Hif1α* deletion in resident cardiac macrophages resulted in the accumulation of Arg1^+^ macrophages by impeding their differentiation.

### The Arg1^+^ differentiation trajectory is protective during MI

**Extended Data Fig. 10 |:**
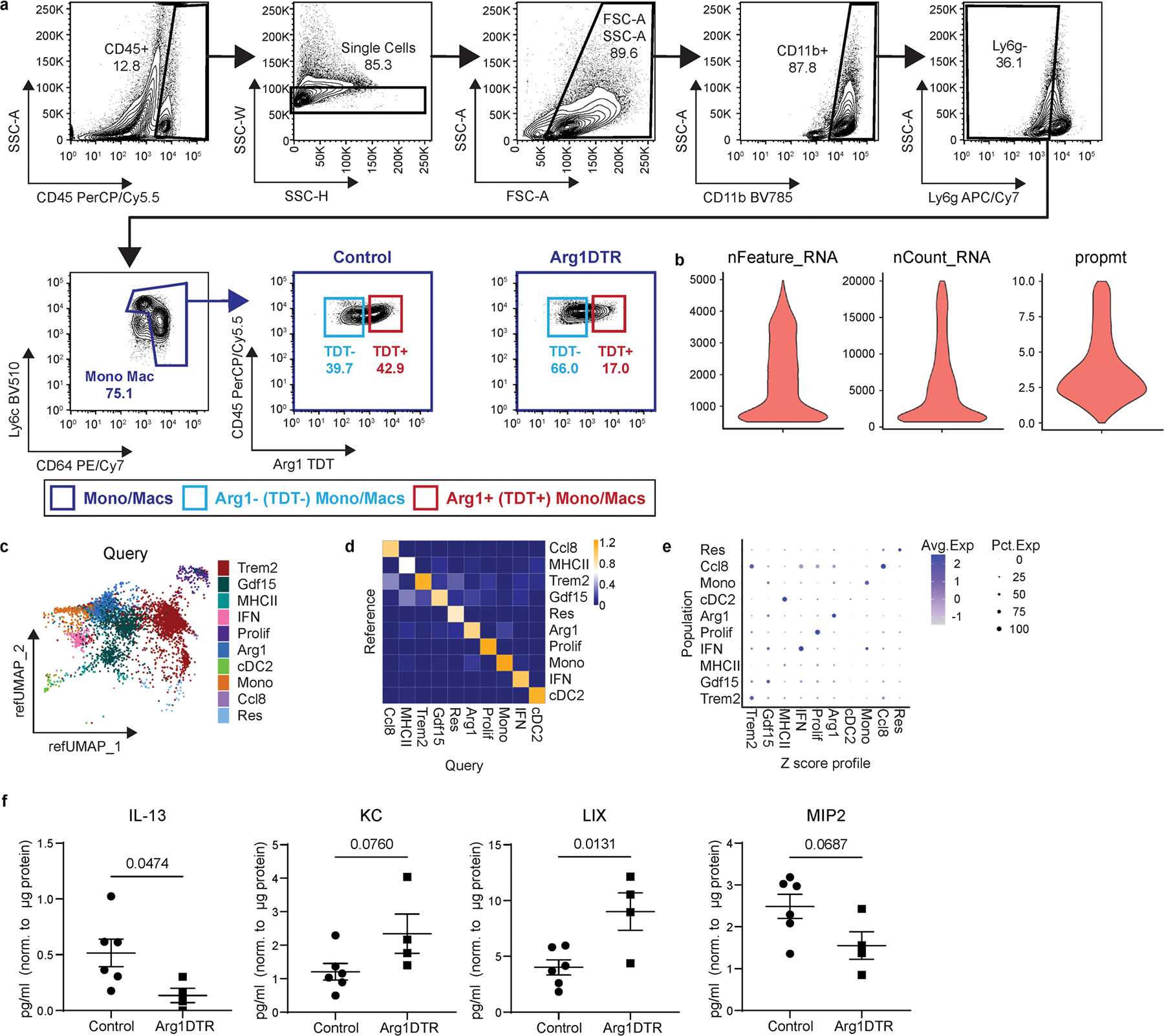
FACS, validation, single cell RNA sequencing, and reference mapping of Arg1DTR mice after MI. **a**, FACS gating strategy for the isolation of monocytes/macrophages/dendritic-like cells and comparison of Arg1-TDT expressing monocytes/macrophages between Control and Arg1DTR mice 3 days after I/R. **b**, Violin plots representing post-quality control scRNAseq data of monocytes/macrophages/dendritic-like cells in Control and Arg1DTR mice 3 days after I/R. Cells with between 500 and 5000 genes (nFeature_RNA), more than 500 and less than 20,000 read counts (nCount_RNA), and percentage of transcripts mapping to mitochondrial genes less than 10 percent (prompt) were used for downstream analysis. **c**, Reference UMAP plot of Control and Arg1DTR scRNAseq data after mapping to reference MI data set. The reference MI scRNAseq data set was the same as the one displayed in Extended Data Fig. 2. **d**, Heat map of mapping scores for each cell population inf the Query (Control and Arg1DTR) plotted against the Reference MI data set. **e**, Z-score profiles from the reference data set (Extended Data Fig. 2f) compared to each subpopulation in the Control and Arg1DTR data set represented as a dot-plot. **f**, IL-13, KC, LIX, and MIP2 protein levels in Control and Arg1DTR mice 3 days after I/R quantified via Luminex. Concentrations (pg/ml) are normalized to the amount of protein in the sample (μg). N = 6 vs 4 (each data point is an individual mouse). P-values are determined using a two tailed t-test assuming equal variance. Data are presented as mean ± SEM.

**Fig. 8 |:**
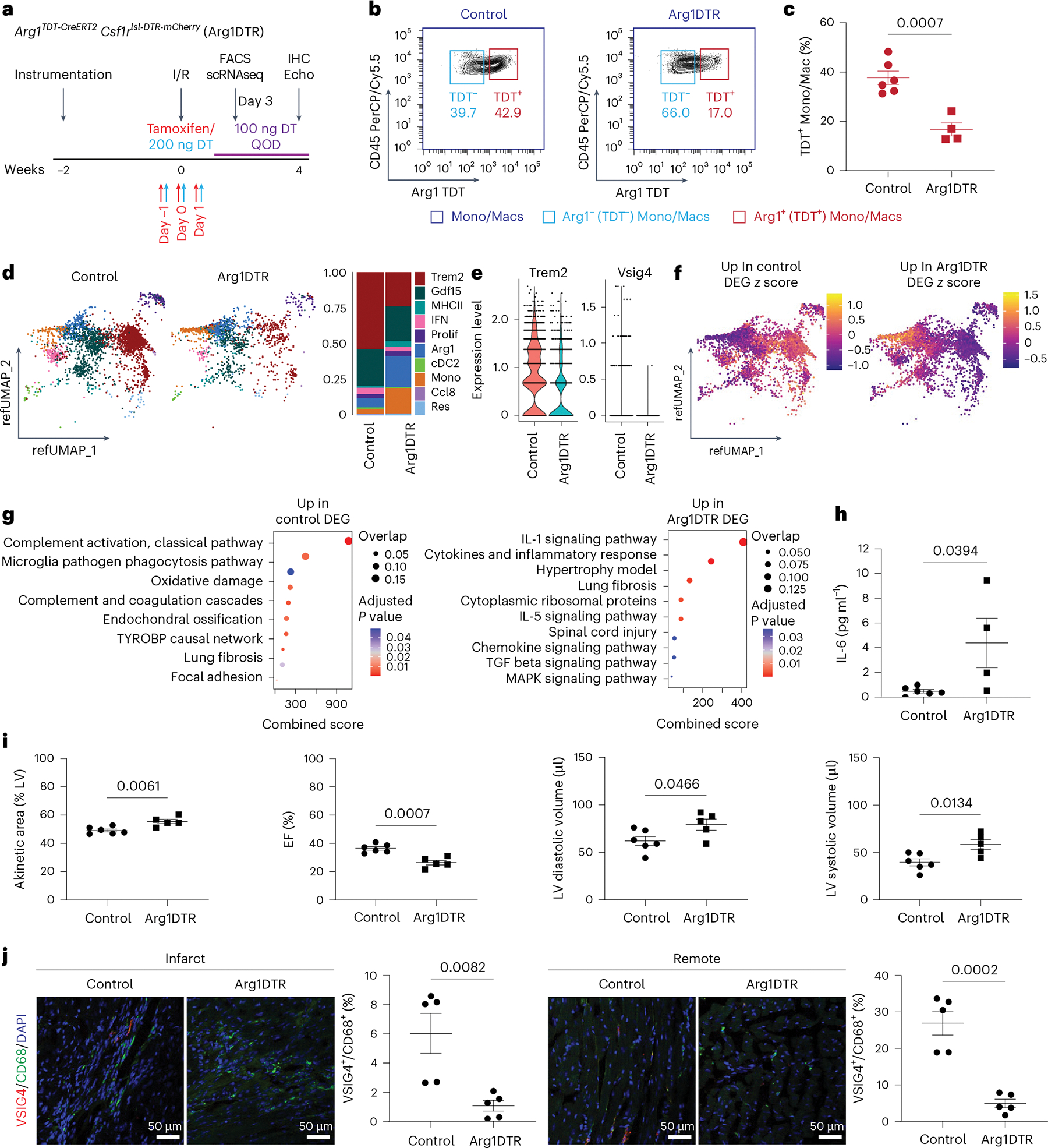
Macrophages downstream of the Arg1^+^ differentiation trajectory are protective following ischemic heart injury. **a**, A schematic of the mouse model of Arg1^+^ macrophage depletion during MI. Arg1DTR mice were given 60 mg kg^−1^ tamoxifen at day −1, 0 and +1 relative to the time of injury to induce DTR expression in Arg1^+^Csf1r^+^ expressing cells. Mice were given 200 ng of DT at day −1, 0 and +1 relative to the time of injury, and afterward 100 ng of DT every other day (QOD) to ablate Arg1^+^Csf1r^+^ cells. Echo, echocardiography. **b**, Representative FACS plot of Arg1-TDT expressing monocytes and macrophages in control and Arg1DTR hearts 3 days after I/R. **c**, Quantification of **b**. **d**, Annotated reference UMAP and stacked bar graph of monocytes/macrophages/dendritic-like cells in control and Arg1DTR mice 3 days after I/R. Data were mapped to a reference MI dataset of monocytes/macrophages/dendritic-like cells 3, 7, 13 and 28 days after I/R. **e**, *Trem2* and *Vsig4* expression in control and Arg1DTR libraries. **f**, *Z*-score profile of DEGs upregulated in control or Arg1DTR plotted on a reference UMAP. **g**, A dot plot of the top upregulated and statistically significant pathways (Enrichr Wikipathways 2019 Mouse) in control and Arg1DTR. **h**, IL-6 protein levels in control and Arg1DTR mice 3 days after I/R quantified via Luminex. Concentrations (in pg ml^−1^) are normalized to the amount of protein in the sample (μg). **i**, Echocardiography quantification of akinetic area as a percentage of the left ventricle, EF, LV diastolic volume and LV systolic volume between control and Arg1DTR mice 4 weeks after I/R. **j**, Representative 20× confocal imaging and quantification of VSIG4 (red), CD68 (green) and DAPI (blue) in the infarct and remote zones of control and Arg1DTR mice 4 weeks after I/R. Data are displayed as the percentage of VSIG4^+^CD68^+^ out of total CD68^+^ cells per 20× field. *P* values were determined using a two tailed *t*-test assuming equal variance. In **c** and **h**, *N* = 4 versus 6; in **i,** *N* = 5 versus 6; and in **j**, *N* = 5 (each data point is an individual mouse). In **c** and **h**–**j**, data are presented as mean ± s.e.m.

We observed increased pathological remodeling, accumulation of Arg1^+^ macrophages and reduction in macrophages downstream of the Arg1^+^ differentiation trajectory in resHif1aKO mice. The remodeling could be due to an increase in the amount of inflammatory Arg1^+^ macrophages or an inability of Arg1^+^ macrophages to differentiate into terminal macrophage populations. To investigate this, we crossed *Arg1*^*TDT-CreERT2*^ mice to *Csf1r*^*lsl-DTR-mCherry*^ mice (Arg1DTR mice) and performed closed-chest I/R^48^. This would cause expression of DTR (diphtheria toxin receptor) under the *Csf1r* promoter in cells that express *Arg1*, thus creating a strategy where Arg1^+^ macrophages and their progeny could be depleted upon administration of tamoxifen and diphtheria toxin (DT). Reduced remodeling would indicate that the resHif1aKO phenotype is caused by an increase in Arg1^+^ macrophages, whereas increased remodeling would indicate it is caused by a decrease in macrophages downstream of the Arg1^+^ differentiation trajectory. Arg1DTR mice were given 60 mg kg^−1^ of tamoxifen and 200 ng of DT at day −1, 0 and +1 relative to the time of injury, and then 100 ng of DT every other day throughout the rest of the experiment (Fig. 8a). Loss of Arg1^+^ macrophages was confirmed 3 days after I/R by FACS (Fig. 8b,c and Extended Data Fig. 10a).

scRNAseq of monocytes/macrophages/dendritic-like cells from Arg1DTR mice 3 days after I/R revealed a reduction in macrophages previously determined to be downstream of Arg1^+^ macrophages (Trem2^+^ macrophages and Vsig4^+^ resident-like macrophages) compared with controls (Fig. 8d,e, Extended Data Fig. 10b–e and Supplementary Table 6). This inability to progress through the Arg1^+^ differentiation trajectory reflects the observation made when resHif1aKO hearts were transplanted to Arg1ZsGr mice (Fig. 7c). Differential expression and pathway analysis between control and Arg1DTR revealed an increase in inflammatory and fibrotic response pathways in Arg1DTR mice (IL-1 signaling, lung fibrosis, TGF-beta signaling and MAPK) largely sourced from monocytes (Fig. 8f,g). Chemokine and cytokine signaling was dysregulated in Arg1DTR hearts 3 days after I/R, including increased IL-6 protein expression (Fig. 8h and Extended Data Fig. 10f).

At 4 weeks after I/R, Arg1DTR mice displayed an increase in akinetic LV area and a decrease in EF (Fig. 8i). IHC of VSIG4^+^CD68^+^ resident-like macrophages confirmed loss of populations downstream of the Arg1^+^ differentiation trajectory (Fig. 8j). The prevalence of LYVE1^+^ and MHC-II^+^ macrophages was not changed between control and Arg1DTR mice, indicating that these populations are derived through alternative monocyte differentiation trajectories (Supplementary Fig. 10). Overall, these results show that cells downstream of the Arg1^+^ differentiation trajectory are necessary for recovery from ischemic injury and the resHif1aKO phenotype, and that the inflammatory gene signature of Arg1^+^ macrophages is secondary to this phenotype.

## Discussion

Here, we provide evidence that *Hif1α* deletion in monocytes and macrophages accelerated LV remodeling following MI and resulted in an increase in a subset of macrophages marked by *Arg1* mRNA and protein expression within the infarct. Arg1^+^ macrophages displayed an inflammatory gene signature, were monocyte-derived and specified early during monocyte to macrophage differentiation during ischemic injury. Mechanistically, Hif1α signaling in resident macrophages served as a cell extrinsic determinant regulating the abundance of Arg1^+^ macrophages. Lineage tracing of Arg1^+^ macrophages in mouse models of I/R showed that Arg1^+^ macrophages differentiated into downstream macrophage subsets including Trem2^+^, Gdf15^+^, MHC-II^hi^ and resident-like macrophages. This Arg1^+^ differentiation trajectory was transcriptionally distinct from other trajectories of monocyte to macrophage differentiation. Finally, we observed that *Hif1α* deletion in resident cardiac macrophages interrupted the progression of monocytes through the Arg1^+^ differentiation trajectory and proper progression of this trajectory is protective in the context of ischemic heart injury.

There are conflicting reports pertaining to the function of Hif1α in myeloid cells following cardiac injury. *Hif1α*^*flox/flox*^ *LysM*^*Cre*^ mice subjected to transverse aortic constriction displayed increased cardiac fibrosis and a reduction in EF^49^. Conversely, a separate study demonstrated that *Hif1α*^*flox/flox*^ *LysM*^*Cre*^ mice displayed decreased infarct size and increased EF following permanent occlusion MI^50^. It is possible that differences in injury models (I/R versus coronary ligation) are a cause: a study that performed I/R on *LysM*^*Cre*^ *Hif1α*^*flox/flox*^ mice found that they display exaggerated myocardial injury, consistent with our data^51^. This indicates that myeloid *Hif1α* may play a role in the reperfusion component of ischemic heart injury. Cellular specificity or temporal control of *Hif1α* deletion may also account for differences in phenotypes. Importantly, *LysM*^*Cre*^ is active during development and throughout the myeloid compartment, including neutrophils^50,52^. While *Cx3cr1* is expressed on antigen-experienced T cells^53^ and may confound our results, the use of pulse-chase tamoxifen dosing and transplant models mitigates this potential limitation.

We found that *Hif1α* deletion in monocytes and macrophages did not influence total macrophage numbers in response to MI, consistent with a previous study^50^. However, scRNAseq revealed that *Hif1α* deletion increased the abundance of Arg1^+^ macrophages and influenced monocyte to macrophage differentiation after MI. *Hif1α* deletion in cardiac resident, but not monocyte-derived, macrophages mediated these phenotypes, indicating that aberrant hypoxia sensing in resident cardiac macrophages has a non-cell autonomous effect on monocyte specification. This finding mirrors the observation that depletion of CCR2^−^ cardiac resident macrophages before MI results in increased Arg1^+^ macrophages^5^. Cardiac resident macrophage death represents an important event following MI that may impact outcomes^15^. However, we observed no difference in resident cardiac macrophages death rates in resHif1aKO mice, suggesting that they are influencing monocyte differentiation via an unknown paracrine mechanism. Our receptor–ligand analysis indicated that resident macrophages signal to multiple cardiac cell types during ischemic injury. Taken together with the observation that Arg1^+^ macrophages begin differentiating when in contact with endothelial cells, the paracrine regulation of resident cardiac macrophages on monocyte differentiation is probably complex and involves multiple cell types.

Transcriptional profiling indicates that Arg1^+^ macrophages displayed enriched expression of genes involved in production of eicosanoids associated with worsened remodeling and inflammatory cytokines^54^. These findings contrast with prior literature arguing that *Arg1* is a marker of the M2 macrophage phenotype^55–57^. It is essential to highlight that the in vivo relevance of the M1/M2 paradigm is uncertain^58^. Consistent with our findings, *Arg1* expressing macrophages are deleterious and contribute to fibrosis in mouse bleomycin lung injury and MI^59,60^.

The functional requirement of Arg1^+^ macrophages in cardiac pathology must be inferred based on the understanding that depleting them will impact downstream macrophage subsets (Trem2^+^, Gdf15^+^, MHC-II^hi^ and resident-like macrophages), which may have differing functions. Indeed, we found that, despite Arg1^+^ macrophages displaying an inflammatory gene signature, their depletion caused a decrease in heart function after MI, an increase in inflammation and a decrease in Trem2^+^ and resident-like macrophages. Together, these results imply that the phenotypes from resHif1aKO mice are largely due to the inability of Arg1^+^ macrophages to fully differentiate into downstream macrophage subpopulations, with the inflammatory gene signature of Arg1^+^ macrophages playing a minor role in this phenotype.

Our findings provide key insights regarding mechanisms of monocyte-derived macrophage differentiation. We showed that Arg1^+^ macrophages are monocyte-derived, are specified within the coronary vasculature before extravasation and differentiate into multiple downstream macrophages subsets. The transcriptional signature and differentiation trajectory of Arg1^+^ macrophages was distinct from interferon-activated macrophages, demonstrating that monocytes commit to unique trajectories upon recruitment to the ischemic heart. MHC-II^hi^ macrophages were not reduced after ablation of the Arg1^+^ differentiation trajectory, and interferon macrophages also displayed a high entropy. Therefore, interferon macrophages to MHC-II^hi^ macrophages may represent a separate monocyte differentiation trajectory, which will require dedicated lineage tracing to investigate. *Hif1α* deletion in resident macrophages halted progression through the Arg1^+^ differentiation trajectory, resulting in accumulation of proinflammatory intermediates. These insights uncover that monocyte fate decisions are initiated within the vascular compartment, and that transitional macrophage subsets exert unique functions and later differentiate into functionally distinct subsets of macrophages.

Our study is not without limitations. The paracrine signaling pathway by which cardiac resident macrophages influence the differentiation of Arg1^+^ macrophages remains to be determined. Additionally, our receptor–ligand analysis predicted that resident cardiac macrophage *Hif1α* KO may impact multiple cardiac cell types. Furthermore, the mechanistic basis by which Arg1^+^ macrophages are specified within the vasculature is unclear. The beneficial effects of macrophages downstream of the Arg1^+^ differentiation trajectory such as Trem2^+^ macrophages and Vsig4^+^ resident-like macrophages should be functionally validated. In this study, we focused on the impact of HIF1α on monocyte differentiation. HIF1α and HIF2α have been shown to have unique roles in myeloid cells in the context of MI, and additional proteins are involved in the hypoxic response, highlighting the importance of investigating the role of other hypoxia response genes on monocyte differentiation^22,50^. Finally, the role of HIF1α in monocyte-derived macrophages in post-MI remodeling requires further investigation.

In conclusion, we demonstrate that hypoxia sensing in cardiac resident macrophages has a protective role following MI and modulates monocyte fate decisions. We identified unique trajectories of monocyte differentiation within the heart and show that hypoxia sensing in resident cardiac macrophages is essential for progression through the Arg1^+^ differentiation trajectory. Collectively, our findings provide insights into the complexity of monocyte differentiation during ischemic heart injury.

## Methods

### Animal models

Animal studies were performed in compliance with guidelines set forth by the National Institutes of Health Office of Laboratory Animal Welfare and approved by the Washington University institutional animal care and use committee. Animals were housed in a controlled environment with a 12 h light–dark cycle, with free access to water and a standard chow diet. Strains used were *Cx3cr1*^*ERT2Cre*20^ (JAX #020940), *Hif1α*^*flox/flox*21^ (JAX #007561), *Rosa26*^*lox-stop-lox tdTomato*44^ (JAX #007914), *Ccr2*^*ERT2Cre*61^, *Arg1*^*YFP*42^ (JAX #015857), *Arg1*^*tdT–CreERT2*^ (ref. 38) (from the Richard M. Locksley laboratory), *Rosa26*^*lox-stop-lox ZsGreen*44^ (JAX #007906), *Csf1r*^*lsl-DTR-mCherry*^ (JAX #024046)^48^ on the C57BL/6 background. Experiments were performed on mice 2–10 months of age and individual experiments contained mice of similar ages in all experimental conditions. Similar numbers of male and female mice were used for experiments. For Cre recombination in all monocytes and macrophages, *Cx3cr1*^*ERT2Cre*^ *Hif1α*^*flox/flox*^ *Rosa26*^*lox-stop-lox tdTomato*^ mice were given tamoxifen food pellets (Envigo TD, 130857) for the entirety of the experiment. For Cre recombination in other lines, mice were given either i.p. injection or gavage of 60 mg kg^−1^ of tamoxifen (Millipore Sigma, T5648) at the indicated frequency and timing. For DTR depletion, mice were given i.p. injection of either 200 ng or 100 ng of DT (Millipore Sigma, D0564). Controls for all experiments (except as specified below) include Cre^−^ mice treated with tamoxifen. For Arg1DTR experiments, controls were Cre^+^ mice and given tamoxifen but not given DT.

### Closed-chest I/R injury

Mice were anesthetized, intubated and mechanically ventilated. The heart was exposed and a suture placed around the proximal left coronary artery. The suture was threaded through a 1 mm piece of polyethylene tubing to serve as the arterial occlude. Each end of the suture was exteriorized through the thorax. The skin was closed and mice were given a 2 week recovery period before induction of ischemia. After 2 weeks, the animals were anesthetized and placed on an Indus Mouse Surgical Monitor system to accurately record electrocardiography during ischemia. Ischemia was induced after anesthetizing the animals. Tension was exerted on suture ends until ST-segment elevation was seen via electrocardiography. Following 90 min of ischemia time, tension was released and the skin was then closed.

### Echocardiography

Mice were sedated with Avertin (0.005 ml g^−1^), and two-dimensional and motion mode images were obtained in the long and short axis views 4 weeks after MI using the VisualSonics770 Echocardiography System. Left diastolic volume, left systolic volume, akinetic area and total LV area were measured using edge detection and tracking software (VivoLab). The EF was calculated as (left diastolic volume – left systolic volume)/left diastolic volume. The akinetic percentage was calculated as the akinetic area/total LV area.

### Syngeneic heart transplant and two-photon intravital imaging

For syngeneic heart transplant of *Cx3cr1*^*ERT2Cre*^ *Hif1α*^*flox/flox*^ into *Arg1*^*tdT-CreERT2*^ *Rosa26*^*lox-stop-lox ZsGreen*^ mice or *Arg1*^*tdT-CreERT2*^ *Rosa26*^*lox-stop-lox ZsGreen*^ into wildtype in the abdominal cavity, an established syngeneic heterotopic cardiac transplant protocol was used^45–47^. Donor mice were perfused with heparinized saline through the infrarenal aorta. Cardiac grafts were collected from donor mice and left in saline on ice for 1 h for cold ischemia. Recipient mice were anesthetized with ketamine (100 mg kg^−1^) and xylazine (10 mg kg^−1^) and maintained with 1–2% isoflurane. The recipient infrarenal aorta and inferior vena cava were connected to the donor ascending aorta and pulmonary artery, respectively.

For *Arg1*^*YFP*^ intravital two-photon imaging following syngeneic heart transplant, established protocols were used^8,62,63^. Cardiac grafts were collected from donor wildtype mice and transplanted into the cervical position of recipient *Arg1*^*YFP*^ mice after 1 h of cold ischemia. The donor ascending aorta and pulmonary artery were connected to the recipient right common carotid artery and right external jugular vein. At 24 h after syngeneic heart transplantation, mice were anesthetized with ketamine (80 mg kg^−1^) and xylazine (10 mg kg^−1^), intubated and connected to a mouse ventilator. Then, 655 nm nontargeted Qdots (Thermo Fisher Scientific) were injected through the intravenous route into recipient mice before imaging. The heart was fixed to an imaging chamber and imaged for 15 min. Time-lapse imaging was performed with a custom-built two-photon microscope running ImageWarp version 2.1 acquisition software (A&B Software). Imaging is terminal and mice were euthanized.

### Histology

Four weeks after MI, mice were euthanized and the hearts perfused with PBS. The hearts were fixed overnight with 4% paraformaldehdye (PFA) in PBS at 4 °C, cut into thirds on the transverse plane, placed into histology cassettes for paraffin embedding and dehydrated in 70% ethanol in water. The hearts were paraffin embedded and cut into 4 μm sections and mounted on positively charged slides. Tissues were stained by Gomori’s trichrome staining (Richard-Allan Scientific, 87020) and WGA staining (Vector Laboratories, RL-1022). Trichrome staining was imaged using a Meyer PathScan Enabler IV. Measurement of infarct length and LV length was performed in Fiji, with data quantification normalizing the length of the infarct to the length of the LV^64^. WGA staining was imaged using the 20× objective of a Zeiss LSM 700 confocal microscope. Four to five 20× fields of the border zone of the infarct were taken per mouse and 12 cross-sectional (not longitudinal) cardiomyocytes per 20× field were selected at random. Measurement of cross-sectional cardiomyocyte area was performed in Fiji. Data are displayed as the average cardiomyocyte cross-sectional area per mouse.

Four days after MI, mice were euthanized and the hearts perfused with PBS. The hearts were incubated in 2% 2,3,5-triphenyltetrazolium chloride (Millipore Sigma, T8877) in PBS for 30 min at 37 °C and then washed in 4% PFA in PBS for 24 h at 4 °C. Pictures were taken with a Zeiss SteREO Discovery v12 stereo microscope. Quantification of the total infarct area normalized to the total heart area was performed with Fiji.

### Immunofluorescence

Mice were euthanized, and the hearts perfused with PBS. The hearts were cut midway on the transverse plane and fixed overnight with 4% PFA in PBS at 4 °C. They were then dehydrated in 30% sucrose in PBS overnight at 4 °C. The hearts were embedded in optimal cutting temperature compound (Sakura, 4583) and frozen at −80 °C for 30 min. Hearts were then sectioned at 15 μm using a Leica cryostat, mounted on positively charged slides, and stored at −80 °C. For staining, the following steps were performed at room temperature and protected from light. Slides were brought to room temperature for 5 min and washed in PBS for 5 min. Sections were then permeabilized in 0.25% Triton X in PBS for 5 min. Sections were then blocked in 5% BSA in PBS for 1 h. Sections were then stained with primary antibody diluted in 1% BSA in PBS for 1 h (rat anti-CD34, Abcam, ab8158 1:200 dilution; rat anti-CD68, BioLegend, 137002 1:400 dilution; rat anti-Ly6g, BD Pharmingen, 551459 1:100 dilution; rabbit anti-Arg1, Cell Signaling Technology, 93668 1:100 dilution; mouse anti-a-SMA conjugated to 488, Cell Signaling Technologies, 46469S 1:100 dilution; rabbit anti-Lyve1, Abcam, ab14917 1:200 dilution; rabbit anti-Vsig4, Abcam, ab252933 1:100 dilution; goat anti-CD64, R&D systems, AF2074 1:100 dilution; rat anti-MHC-II BioLegend, 107602 1:100 dilution; rat anti-KI67, Thermo Fisher Scientific, 14–5698-82 1:100 dilution). Sections were then washed three times for 5 min each with PBS–Tween. After washing, the appropriate secondary antibodies were added diluted in 1% BSA in PBS for 1 h at a 1:1,000 dilution (goat anti-Rat 488, Thermo Fisher Scientific, a11006; goat anti-Rat 647, Thermo Fisher Scientific, a21247; donkey anti-Rabbit 647, Abcam, ab150075; chicken anti-Rabbit 647, Thermo Fisher Scientific, a21443; goat anti-Rabbit 555, Thermo Fisher Scientific, a21428; donkey anti-Rat 488, Thermo Fisher Scientific, a21208; donkey anti-Rat 647, Thermo Fisher Scientific, a78947; donkey anti-Goat 555, Thermo Fisher Scientific, a21432). Slides were again washed three times for 5 min each with PBS–Tween and then mounted with 4,6-diamidino-2-phenylindole (DAPI) mounting media (Millipore Sigma, F6057) and cover slipped. Slides were stored at 4 °C protected from light and imaged within 1 week. For immunofluorescence staining of paraffin embedded tissues collected 4 weeks after MI, the OPAL 4-color manual IHC kit (Akoya Biosciences, NEL810001KT) was used. Primary antibodies used were anti-CD34 (Abcam, ab8158 1:200 dilution) and anti-a-SMA (Invitrogen, 14–9760-82 1:500 dilution).

Slides were imaged using the 20× or 40× objective of a Zeiss LSM 700 confocal microscope using Zeiss Zen Black software. Visualization of TDT and ZsGreen was from the endogenous signal. For quantification, four to five 20× fields were taken per mouse of either the remote zone, border zone, infarct zone or myocardium, as indicated. Quantification of cell numbers, area percentages, or a-SMA^+^ vessels were performed in Fiji or Zeiss Zen, and data are displayed as the average of each 20× field per mouse.

### Flow cytometry

Hearts were perfused with PBS, weighed, minced and digested for 45 min at 37 °C in Dulbecco’s modified Eagle medium (DMEM) (Gibco, 11965–084) containing 4,500 U ml^−1^ collagenase IV (Millipore Sigma, C5138), 2,400 U ml^−1^ hyaluronidase I (Millipore Sigma, H3506) and 6,000 U ml^−1^ DNAse I (Millipore Sigma, D4527). Enzymes were deactivated with Hanks’ balanced salt solution containing 2% FBS and 0.2% BSA and filtered through 40 μm strainers. Cells were incubated with ammonium-chloride-potassium (ACK) lysis buffer (Gibco, A10492–01) for 5 min at room temperature. Cells were washed with DMEM and resuspended in 100 μl of FACS buffer (PBS containing 2% FBS and 2 mM EDTA).

Approximately 100 μl of blood was collected by cheek bleed into a tube containing 20 μl of EDTA (Corning, 46–034-CI). Cells were incubated with ACK lysis buffer for 15 min at room temperature, washed and ACK lysed a second time. Cells were then washed with DMEM and resuspended in 100 μl of FACS buffer.

Cells were incubated in a 1:200 dilution of fluorescence-conjugated monoclonal antibodies for 30 min at 4 °C. This dilution was used throughout the study for both FACS analysis and sorting. Samples were washed in FACS buffer and resuspended in a final volume of 250 μl FACS buffer. Flow cytometry and sorting was performed using a BD FACS melody with BD FACS Chorus software.

For FACS analysis of monocytes, neutrophils and macrophages in macHif1aKO mice, the antibodies used were CD45 PerCP/Cy5.5 (BioLegend, 103132), CD64 PE/Cy7 (BioLegend, 139313), Ly6g FITC (BioLegend, 127606) and Ly6c APC (BioLegend, 128016).

For sorting of monocytes and macrophages in macHif1aKO mice for scRNAseq, the antibodies used were CD45 PerCP/Cy5.5 (BioLegend, 103132), Ly6g FITC (BioLegend, 127606), Ly6c APC (BioLegend, 128016), DAPI (BD Pharmingen, 564907) and CD64 PE/Cy7 (BioLegend, 139313).

For sorting of blood monocytes in monoHif1aKO mice for qPCR, the antibodies used were CD45 PerCP/Cy5.5 (BioLegend, 103132), CD11b BV421 (BioLegend, 101235), CD64 PE/Cy7 (BioLegend, 139313), Ly6g APC/Cy7 (BioLegend, 127623), Ly6c BV510 (BioLegend, 128033) and CD115 APC (BioLegend, 135509).

For sorting and FACS analysis of monocytes and macrophages in *Arg1*^*tdT-CreERT2*^, Arg1–YFP, Arg1ZsGr, control>ZsGr and Hif1a>ZsGr mice, the antibodies used were CD45 PerCP/Cy5.5 (BioLegend, 103132), CD11b BV421 (BioLegend, 101235), Ly6g APC/Cy7 (BioLegend, 127623), Ly6c BV510 (BioLegend, 128033) and CD64 PE/Cy7 (BioLegend, 139313).

For sorting and FACS/waterfall analysis of monocytes and macrophages in *Arg1*^*tdT-CreERT2*^ *Csf1r*^*lsl-DTR-mCherry*^ mice 3 days after I/R, the antibodies used were CD45 PerCP/Cy5.5 (BioLegend, 103132), CD11b BV785 (BioLegend, 101243), Ly6g APC/Cy7 (BioLegend, 127623), Ly6c BV510 (BioLegend, 128033), MHC-II BV421 (BioLegend, 107631) and CD64 PE/Cy7 (BioLegend, 139313).

For intravascular staining, 200 μl of CD45 APC (BioLegend 103112) was injected intravascularly at a concentration of 5 μg ml^−1^ in PBS 5 min before tissue collection. Hearts were then perfused with 10 ml of PBS.

For intracellular staining of Arg1–YFP, the BioLegend Cyto-Fast Fix/Perm Buffer Set (BioLegend, 426803) was used following staining for surface markers, and intracellular staining with GFP–PE (BioLegend, 338003) was performed.

For assessment of resident cardiac macrophage death in resHif1aKO mice 1 day after I/R, a Cytek Aurora was used using Cytek SpectroFlo software. Antibodies used were MHC-II APC/Cy7 (BioLegend, 107628), CCR2 BV421 (BioLegend, 150605), CD64 APC (BioLegend, 139306), Zombie Aqua (BioLegend, 77143), Ly6c PE/Cy7 (BioLegend, 128017), Ly6g FITC (BioLegend, 127606), CD45 Percp/Cy5.5 (BioLegend, 103132) and CD11b BV785 (BioLegend, 101243).

### scRNAseq analysis

Monocytes and macrophages from macHif1aKO and control mice were sorted 5 days after I/R. scRNAseq libraries were generated using the 10X Genomics 5′v1 platform. Libraries were sequenced using a NovaSeq at the McDonnel Genome Institute. Sequencing reads were aligned to the GRcm38 transcriptome using CellRanger (v3.1.0) from 10X Genomics.

ZsGr^−^ and ZsGr^+^ monocytes and macrophages were sorted from Arg1ZsGr mice 2 and 30 days after I/R. scRNAseq libraries were generated using the 10X Genomics 5′v1 platform. Sequencing reads were aligned to the mm10–2020-A transcriptome using CellRanger (v7.1.0)

ZsGr^−^ and ZsGr^+^ monocytes and macrophages were sorted from control>ZsGr and Hif1a>ZsGr mice 5 days after syngeneic heart transplant. Libraries were generated using the 10X 3′v3.1 platform. Sequencing reads were aligned to the mm10–2020-A transcriptome using CellRanger (v7.1.0).

Monocytes and macrophages from control and Arg1DTR mice were sorted 3 days after I/R. Libraries were generated using the 10X 3′v3.1 platform. Sequencing reads were aligned to the mm10–2020-A transcriptome using CellRanger (v8.0.0).

Downstream data processing was performed in the R package Seurat (v4)^65^. SCtransform was used to scale and normalize the data for RNA counts. Quality control filters of genes/cell, mitochondrial reads/cell and read counts/cell were applied as indicated (Extended Data Figs. 2b, 6d, 8c and 10b). Principal component analysis was used for dimensionality reduction, data were clustered at multiple resolutions and Uniform Manifold Approximation and Projection (UMAP) projections were generated for data visualization. Harmony integration was used for Arg1ZsGr I/R data to account for batch effect from different time points^66^. Contaminating populations of neutrophils and nonmyeloid cells were excluded from analysis. Annotation of cell populations in I/R datasets was achieved by using label transfer in Seurat, mapping the data to a reference dataset of myeloid cells present in the mouse heart 3, 7, 13 and 28 days after MI^29,30^. Differential gene expression of upregulated genes in each subpopulation was generated in Seurat using FindAllMarkers with a minimum fraction (min.pct) of 0.1 and a log fold change threshold of 0.25. Differential gene expression was performed through Seurat. Genes from FindAllMarkers and FindMarkers were used as input for Wikipathways 2019 Mouse analysis and ChEA 2016 transcription factor analysis in EnrichR^67–71^. Gaussian kernel density estimation plots were generated using the Python package Scanpy (v1.8.1)^72^.

For analysis of publicly available datasets GSE119355, E-MTAB-7376, GSE135310, GSE197441 and GSE197853, Seurat v5.0.1 was used^15,23–25,73^. Quality control filters of 200–6,000 genes per cell, less than ten mitochondrial reads per cell and less than 40,000 read counts per cell were applied. To be in accordance with previous integrated analysis of these data, log normalization was used instead of SCTransform. Previously published scripts were used to assist with data processing^23^. Harmony integration was performed based on the library that the data came from to account for batch effect from different experiments. Contaminating populations of neutrophils and nonmyeloid cells were excluded from analysis. Enriched genes identified from a previously published analyses of these data were used to assist with annotation^23^. Comparison of macHif1aKO data to publicly available datasets was achieved by using label transfer in Seurat and by calculating the Arg1^+^ macrophage gene signature *z*-score (*Arg1*, *Fn1*, *Cxcl1* and *Tppp3*).

### snRNAseq analysis

Hearts from six control and six resHif1aKO mice 3 days after I/R were perfused with PBS and minced, and nuclei were isolated using the 10X Genomics Nuclei Isolation kit with RNase inhibitor (PN-1000494). The resulting single-nuclei preparations were pooled based on experimental condition and were stained with a 1:200 dilution of DRAQ5 (Thermo Fisher Scientific, 65–0880-96). DRAQ5^+^ nuclei were sorted into 300 μl of wash buffer with RNase Inhibitor. Two snRNAseq libraries per condition were generated using the 10X Genomics 5′v2 platform. Libraries were sequenced using a NovaSeq at the McDonnel Genome Institute. Sequencing reads were aligned to the mm10–2020-A transcriptome using CellRanger (v8.0.0) from 10X Genomics.

Downstream data processing was performed as described for scRNAseq analysis. After quality control filters were applied (Extended Data Fig. 5b), scrublet v0.2.3 was employed to identify doublets^74^. Nuclei with a scrublet score higher than 0.25 were excluded from downstream analysis. After initial clustering and annotation, cardiomyocyte + epicardium; endocardium + endothelium; fibroblast, myeloid, NKT (natural killer and T-cells) and pericyte + SMC populations were subset and individually reclustered to remove contaminating populations, and then recombined and reclustered with adipocyte, B cell, lymphatic, neuron, neutrophil and pDC (plasmacytoid dendritic cell) populations to generate a final dataset. For receptor–ligand analysis on scRNAseq macHif1aKO and snRNAseq resHif1aKO data, CellChat v2 was used^75^.

### Palantir trajectory analysis

The Palantir python package was used for trajectory analysis (v1.0.0 to v1.1)^37^. Before inputting data to Palantir, cell cycle regression was performed using Seurat^76^. Monocytes were used as the early and starting cell type for Palantir. Highly variable gene analysis was not used. The eigenvectors used were determined by the eigengap. The number of waypoints used was 500. The option to use the early cell as the start cell was used. The output pseudotime/entropy values, terminal state probabilities and force-directed layout (FDL) coordinates for each cell was used to generate plots in R.

### QPCR

For assessment of recombination efficiency of the *Hif1α*^*flox/flox*^ allele, 100,000 monocytes and macrophages from control and macHif1aKO or monoHif1aKO mice hearts were sorted into RLT buffer 5 days after closed-chest I/R. Approximately 50,000 monocytes were sorted from the blood of control and monoHif1aKO mice 24 h after tamoxifen administration. RNA was isolated using the RNeasy Micro kit (Qiagen, 74004). cDNA was generated using the iScript Reverse Transcription Supermix (Bio-Rad, 1708841) kit. qPCR reactions were prepared with PowerUP SYBR Green Master Mix (Thermo Fisher Scientific, A25742). Reference control *36b4* primers used were as follows: forward: ATCCCTGACGCACCGCCGTGA, reverse: TGCATCTGCTTGGAGCCCACGTT. *Hif1α* primers used were as follows: forward: TTCTGTTATGAGGCTCACCATC, reverse: TCTGTGCCTTCATCTCATCTTC. Primers were purchased from Integrated DNA Technologies. qPCR was performed using QuantStudio 3 (Thermo Fisher Scientific). *Hif1α* expression was normalized to *36b4* expression.

### Cytokine and chemokine assay

Heart homogenate from Arg1DTR hearts was collected by combining the heart with 500 μl of PBS and two steel beads (Qiagen, 69989) into Safe-Lock tubes (Eppendorf, 022363344). The hearts were homogenized in a TissueLyser LT (Qiagen) at 50 Hz for 5 min. Homogenates were spun down at 5,000 rpm for 10 min and supernatant was used for protein quantification. Protein concentration was measured by bicinchoninic acid (Abcam, ab207003). Cytokine and chemokine protein contents in heart homogenates were quantified by Luminex multianalyte technology (Millipore) according to the manufacturer’s instructions. The samples were read using a Bio-Plex 200 instrument. The results were analyzed using Bio-Plex software version 6.1 (Bio-Rad). Finally, the values were normalized to amount of protein used according to bicinchoninic acid. Out-of-range values were considered as zero.

### Statistical analysis

All experiments were powered to identify statistically significant differences and included multiple independent biological replicates. For analysis of echocardiography, histology, immunofluorescence and FACS, a two tailed *t*-test assuming equal variance was performed in Excel or Prism. For statistical comparison between more than two conditions, an ordinary one-way analysis of variance (ANOVA) comparing the mean of each column with the mean of every other column using Tukey’s multiple comparisons test was performed in Prism or the R package multcomp. *P* < 0.05 was a priori considered statistically significant. Data were plotted using Prism software. As indicated in figure legends in graphs of quantifications, each data point represents an individual mouse (biological replicate). For qPCR, two technical replicates per biological replicate were performed. Graphs of quantifications show the mean and s.e.m.

## Extended Data

## Supplementary Material

supplemental data

supplemental tables

**Supplementary information** The online version contains supplementary material available at https://doi.org/10.1038/s44161-024-00553-6.

## Data Availability

scRNAseq and snRNAseq data are available on the Gene Expression Omnibus (GSE251991). Publicly available datasets used in this study are available at EnrichR/Wikipathways/ChEA (https://mayyanlab.cloud/Enrichr/) and PMID 30582448 (available on https://www.immgen.org/), GSE119355, E-MTAB-7376, GSE135310, GSE197441 and GSE197853. Source data are provided with this paper.
