# Supplementary material for: Hypoxia sensing in resident cardiac macrophages regulates monocyte fate specification following ischemic heart injury
Source: Nat Cardiovasc Res. Author manuscript; Available in PMC 2025 Dec 11. (PMC12696718; doi:10.1038/s44161-024-00553-6)
Supplement: supplemental data [file NIHMS2121066-supplement-supplemental_data.pdf]

nature cardiovascular research

Supplementary information https://doi.org/10.1038/s44161-024-00553-6

Hypoxia sensing in resident cardiac macrophages regulates monocyte fate specification following ischemic heart injury

In the format provided by the authors and unedited

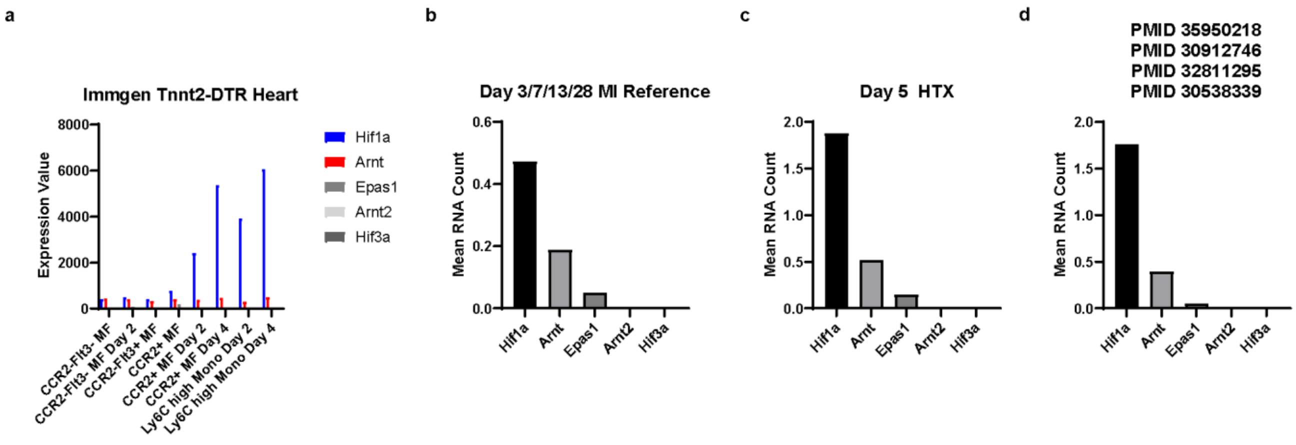

Supplementary Figure 1. Expression of Hypoxia Response Genes in Cardiac Macrophages. a, Immgen mRNA expression data of *Hif1a*, *Arnt*, *Epas1*, *Arnt2*, and *Hif3a* in $CCR2^-$ cardiac macrophages, $CCR2^+$ cardiac macrophages, and $Ly6c^{high}$ Monocytes in a Tnnt2-DTR cardiomyocyte ablation model of heart injury from PMID 30582448 (available from immgen.org). **b,** Mean RNA counts across all cells of *Hif1a*, *Arnt*, *Epas1*, *Arnt2*, and *Hif3a* in a reference MI scSEQ data set of monocytes/macrophages/dendritic-like cells 3, 7, 13, and 28 days after I/R. **c,** Mean RNA counts across all cells of *Hif1a*, *Arnt*, *Epas1*, *Arnt2*, and *Hif3a* in the day 5 post syngeneic heart transplant data set of monocytes/macrophages/dendritic-like cells presented in Figure 6 and Figure 7. **d,** Mean RNA counts across all cells of *Hif1a*, *Arnt*, *Epas1*, *Arnt2*, and *Hif3a* in monocytes/macrophages/dendritic-like cells from publicly available scSEQ data sets of mouse MI 0, 1, 3, 5, 7, and 11 days after injury (PMID 35950218, PMID 30912746, PMID 32811295, PMID 30538339).

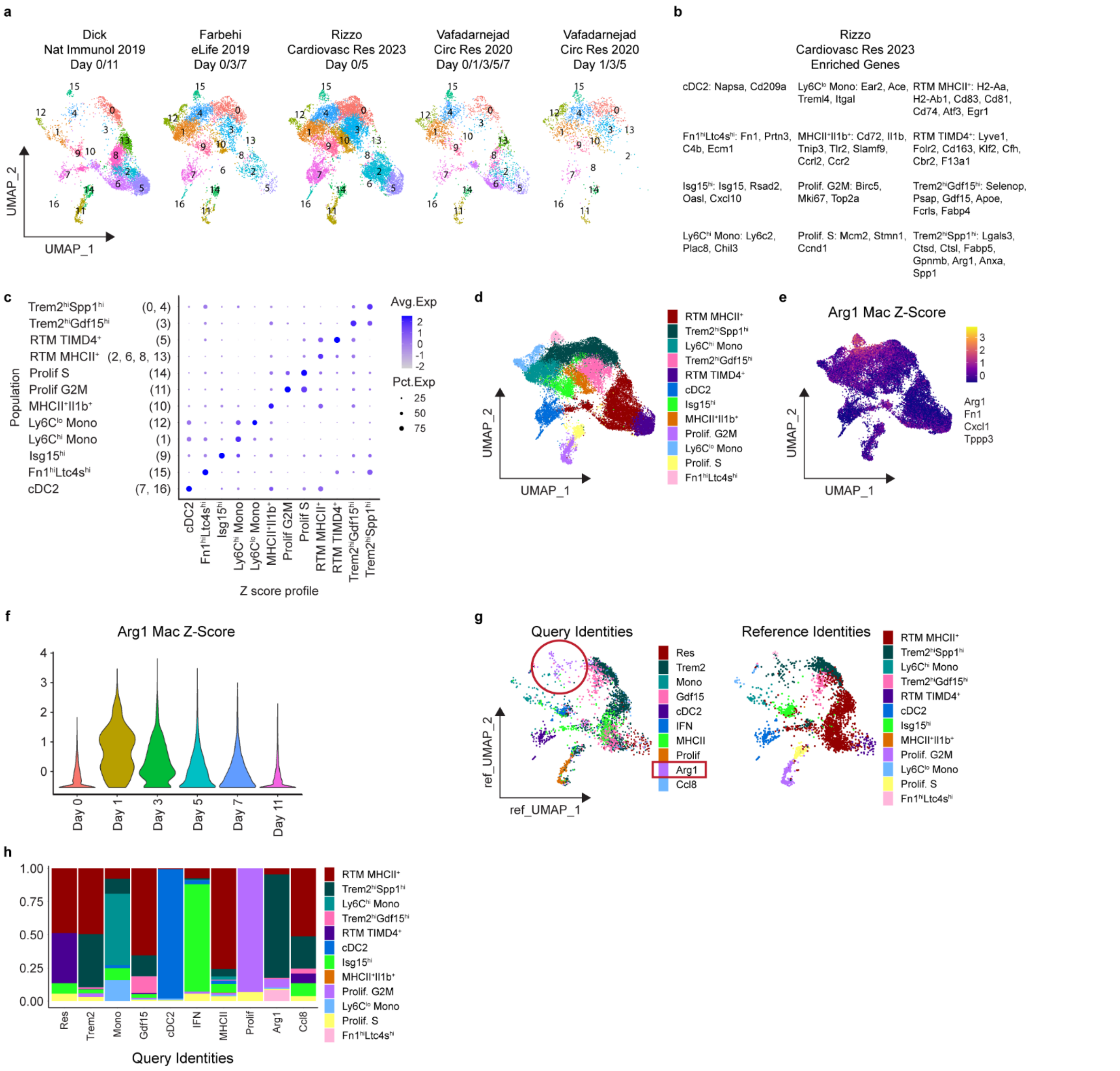

Supplementary Figure 2. Arg1 expressing macrophages are represented early after injury in publicly available datasets of mouse MI. a, UMAP projection of harmony integrated MI data sets from PMID 30538339, PMID 30912746, PMID 35950218, and PMID 32811295. **b,** Marker genes of subpopulations identified by *Rizzo et al.* used to determine the subpopulation identities of a. **c,** Z-scores from b compared to each subpopulation identified. The Y-axis shows the subcluster in parentheses after the corresponding subpopulation it was determined to be. **d,** UMAP projection of harmony integrated MI data sets from a grouped by annotations. **e,** Z-score profile of Arg1^{+} macrophages (*Arg1*, *Fn1*, *Cxcl1*, *Tpp3*) plotted on UMAP. **f,** Violin plot of Arg1^{+} macrophage Z-score split between timepoints. **g,** Reference UMAP of macHif1aKO data mapped to data from d, with cells grouped by either their original (query) identity or mapped (reference) identity. **h,** Stacked bar graph representing the proportion of reference cell identities split by the original query identities.

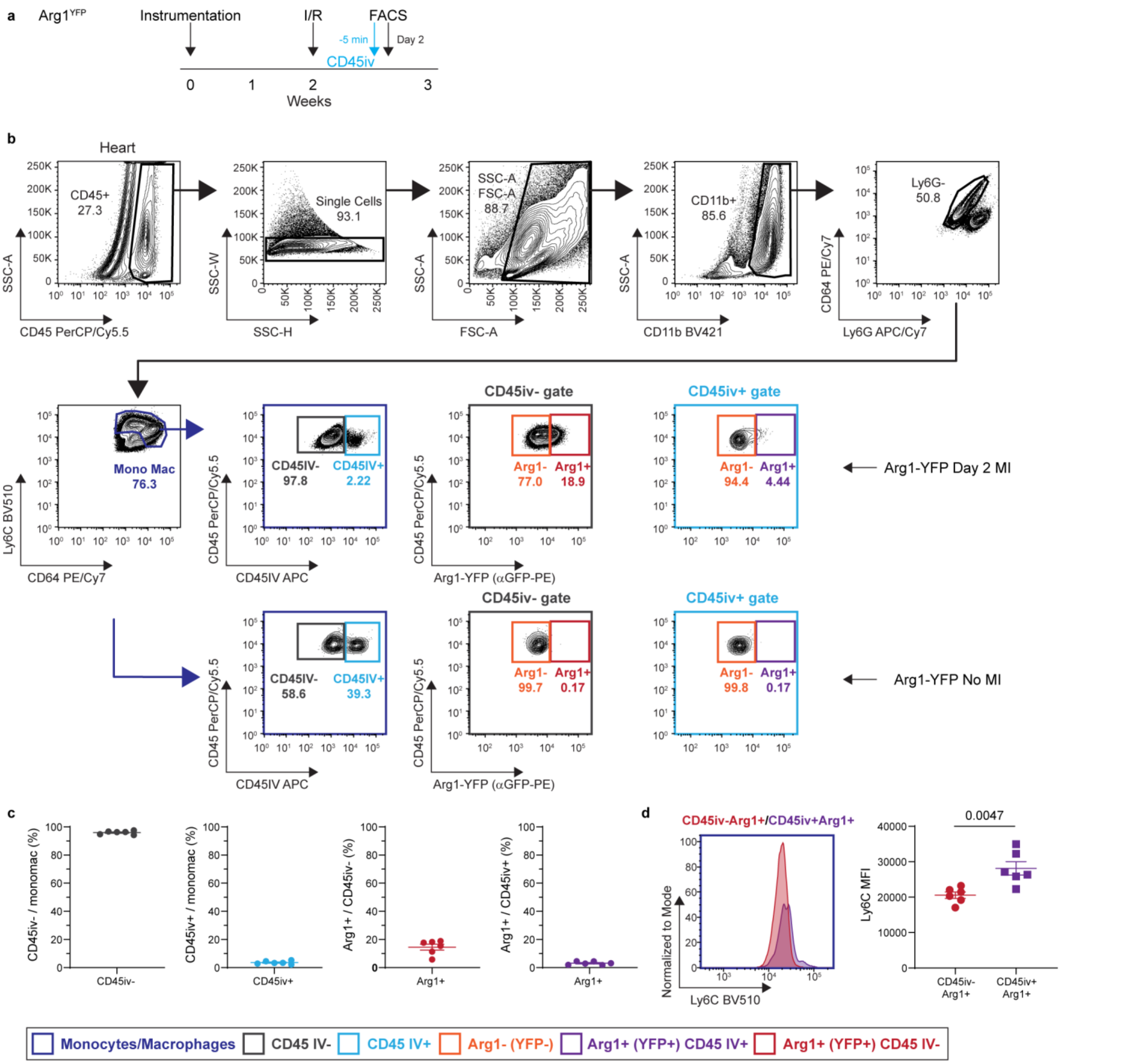

Supplementary Figure 3. Intravascular staining FACS of *Arg1*^{YFP} mice 2 days after MI a, Schematic of intravascular FACS in *Arg1*^{YFP} mice 2 days after I/R. Mice were injected with a CD45 antibody 5 minutes prior to tissue collection. **b,** FACS gating strategy for intravascular staining in the heart of *Arg*^{YFP} mice 2 days after I/R or no injury. Cells were gated for leukocytes (SSC-A vs CD45), single cells (SSC-W vs SSC-H), debris exclusion (SSC-A vs FSC-A), myeloid cells (SSC-A vs CD11b), neutrophil exclusion (CD64 vs Ly6G) and monocytes/macrophages (Ly6C vs CD64). Intravascular (CD45IV+) and extravascular cells (CD45IV-) were identified (CD45 vs CD45IV). Arg1^{+} cells were identified via intracellular anti-GFP staining in the CD45iv- and CD45iv+ gate (CD45 vs Arg1-YFP (αGFP)). **c,** Quantification of populations of interest from Day 2 MI mice in b. **d,** Representative histogram of Ly6C staining between CD45iv^{-}Arg1^{+} cells and CD45iv^{+}Arg1^{+} cells. Quantification of Ly6C mean fluorescence intensity (MFI) between CD45iv^{-}Arg1^{+} cells and CD45iv^{+}Arg1^{+} cells. P-values are determined using a two tailed t-test assuming equal variance. N = 6 (each data point represents an individual mouse).

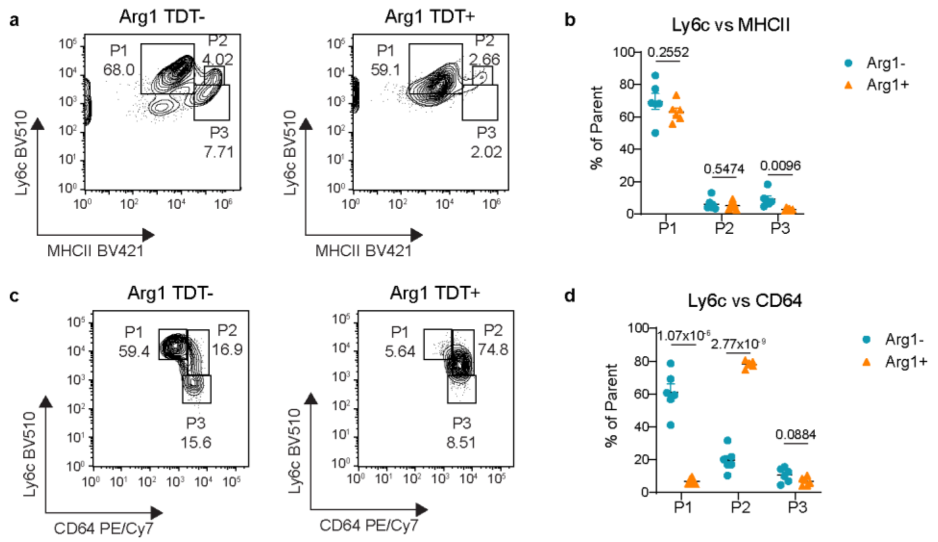

Supplementary Figure 4. Monocyte-waterfall analysis of Arg1+ macrophages 3 days after MI. a, Monocyte-waterfall Ly6c/MHCII plot of Arg1-TDT^{-} and Arg1-TDT^{+} monocytes/macrophages. P1, P2, and P3 represent the developmental trend of monocytes differentiating into macrophages. **b,** Quantification of data in a, representing the amount of Arg1-TDT^{-} and Arg1-TDT^{+} monocytes/macrophages in each monocyte to macrophage differentiation population (P1/P2/P3) as a percentage of total Arg1-TDT^{-} or Arg1-TDT^{+} monocytes/macrophages. N = 6 individual mice. **c,** Monocyte-waterfall Ly6c/CD64 plot of Arg1-TDT^{-} and Arg1-TDT^{+} monocytes and macrophages, with P1/P2/P3 representing developmental stages of monocyte to macrophage differentiation. **d,** Quantification of data in d. Arg1-TDT^{-}/Arg1-TDT^{+} cells were first gated on CD45+, CD11b+, Ly6g+, Ly6c/CD64+. P-values are determined using a two tailed t-test assuming equal variance. N = 6 individual mice.

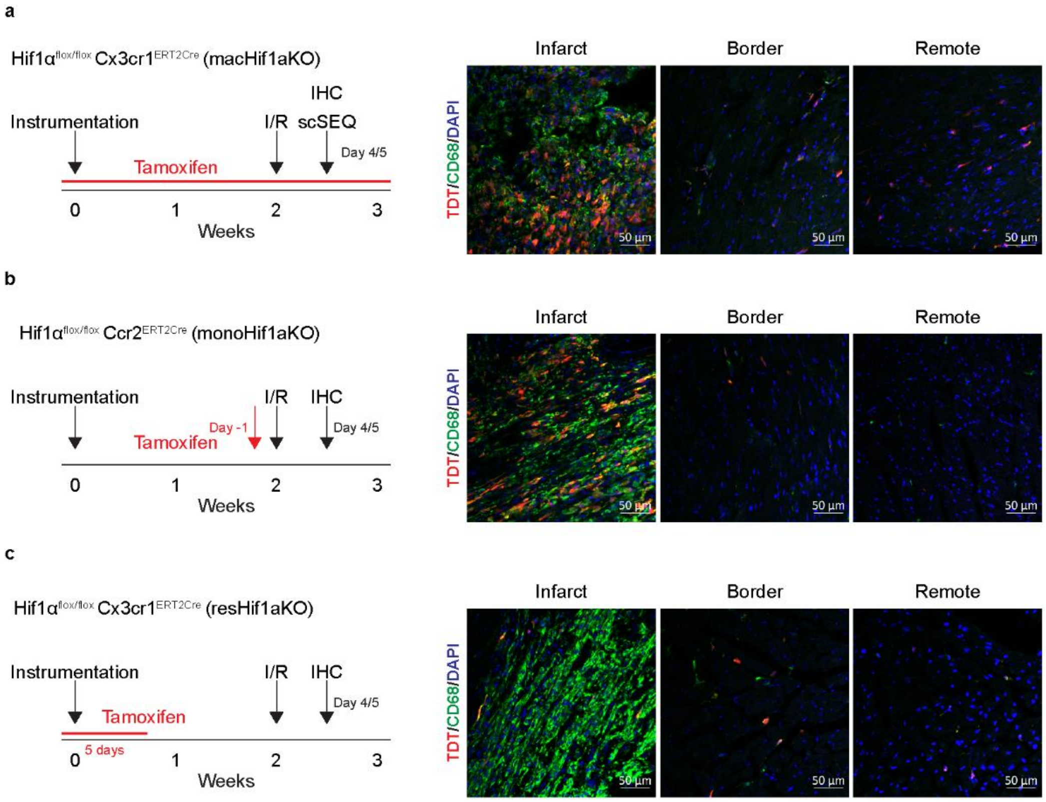

Supplementary Figure 5. Tamoxifen strategies to induce Cre recombination in all monocytes and macrophages, recruited monocytes and macrophages, or cardiac resident macrophages. a, Schematic of experimental design and representative 20x confocal images in the infarct, border, and remote zone when *Hif1α* is knocked out in all monocytes and macrophages during closed chest I/R (macHif1aKO). **b,** Schematic of experimental design and representative 20x confocal images in the infarct, border, and remote zone when *Hif1α* is knocked out in monocytes and monocyte derived macrophages during closed chest I/R (monoHif1aKO). **c,** Schematic of experimental design and representative 20x confocal images in the infarct, border, and remote zone when *Hif1α* is knocked out in resident macrophages during closed chest I/R (resHif1aKO). Endogenous TDT signal is in red, CD68 IHC staining is in green, and DAPI is in blue. Results were representative of that obtained from at least 2 prior experiments.

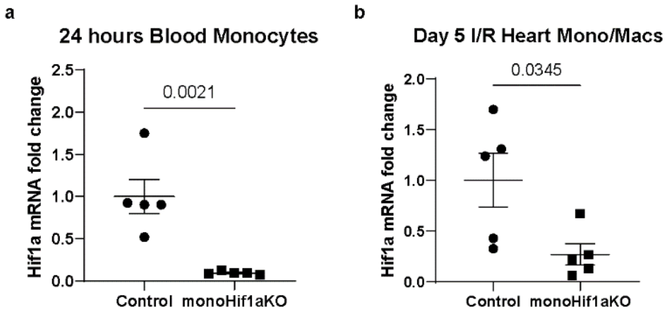

Supplementary Figure 6. Cre recombination induced knockout of *Hif1a* in monocytes and monocyte-derived macrophages. a, qPCR of *Hif1a* mRNA expression in blood monocytes of Control and monoHif1aKO mice 24 hours after IP injection of 60 mg/kg of tamoxifen. Monocytes were gated as CD45+, Ly6g-, Cd11b+, Ly6c+, CD115+. **b,** qPCR of *Hif1a* mRNA expression in sorted monocytes and macrophages from Control and monoHif1aKO mice 5 days after I/R. N = 5 (each data point represents an individual mouse). P-values are determined using a two tailed t-test assuming equal variance.

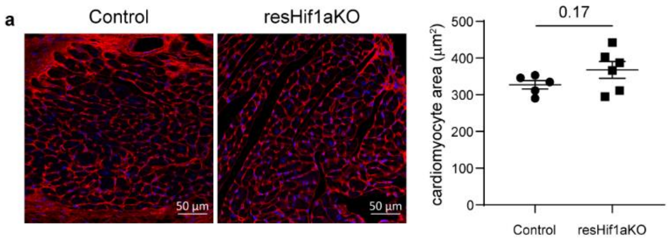

Supplementary Figure 7. WGA staining in resHif1aKO mice a, Representative WGA staining of the border zone of the infarct of Control and resHif1aKO hearts 4 weeks after I/R. Quantification of the average cardiomyocyte area was determined from WGA staining. P-values are determined using a two tailed t-test assuming equal variance. N = 5 vs 6 (each data point represents an individual mouse).

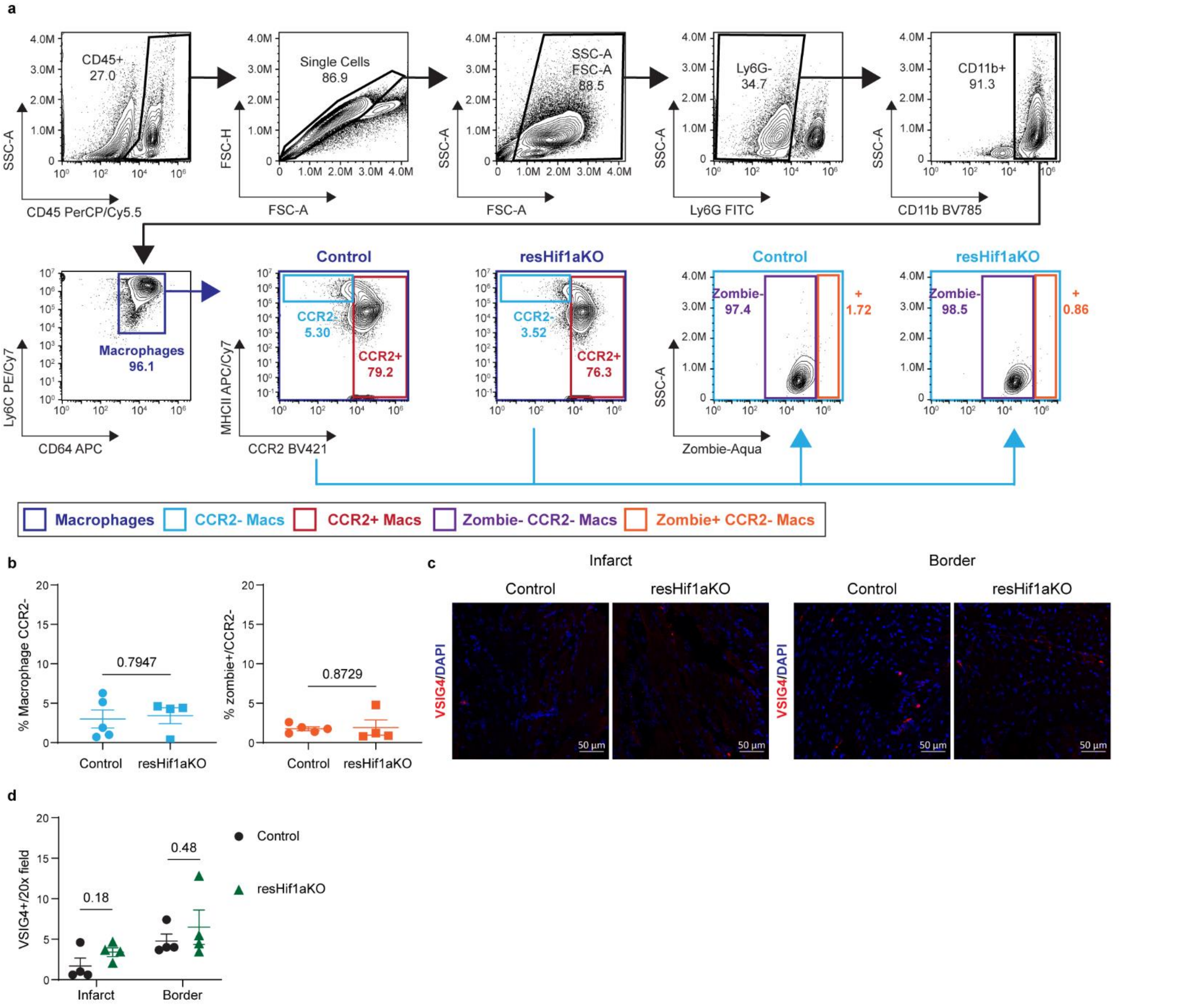

Supplementary Figure 8. ***Hif1α*** **deletion in resHif1aKO mice does not affect resident cardiac macrophage cell death after MI. a,** FACS gating strategy for resHif1aKO mice 1 day after I/R. Cells were gated for leukocytes (SSC-A vs CD45), single cells (FSC-H vs FSC-A), debris exclusion (SSC-A vs FSC-A), neutrophil exclusion (SSC-A vs Ly6G), myeloid cells (SSC-A vs CD11b), macrophages (Ly6C vs CD64), and resident (CCR2$^-$) vs recruited (CCR2$^+$) macrophages (MHCII vs CCR2). Cell death rate in CCR2$^-$ macrophages was determined by Zombie staining (SSC-A vs Zombie-Aqua). **b,** Quantification of cell types of interests in a. The percentage of macrophages that are CCR2$^-$ is displayed, followed by the percentage of CCR2$^-$ macrophages that are Zombie$^+$. N = 4-5 (each data point represents an individual mouse). **c,** Representative 20x confocal images of resident macrophage marker VSIG4 (red) and DAPI (blue) IHC in the infarct and border zones of Control and resHif1aKO mice 1 day after I/R. **d,** Quantification of IHC in c, displayed as the total number of VSIG4+ cells per 20x field. N = 4 individual mice. P-values are determined using a two tailed t-test assuming equal variance.

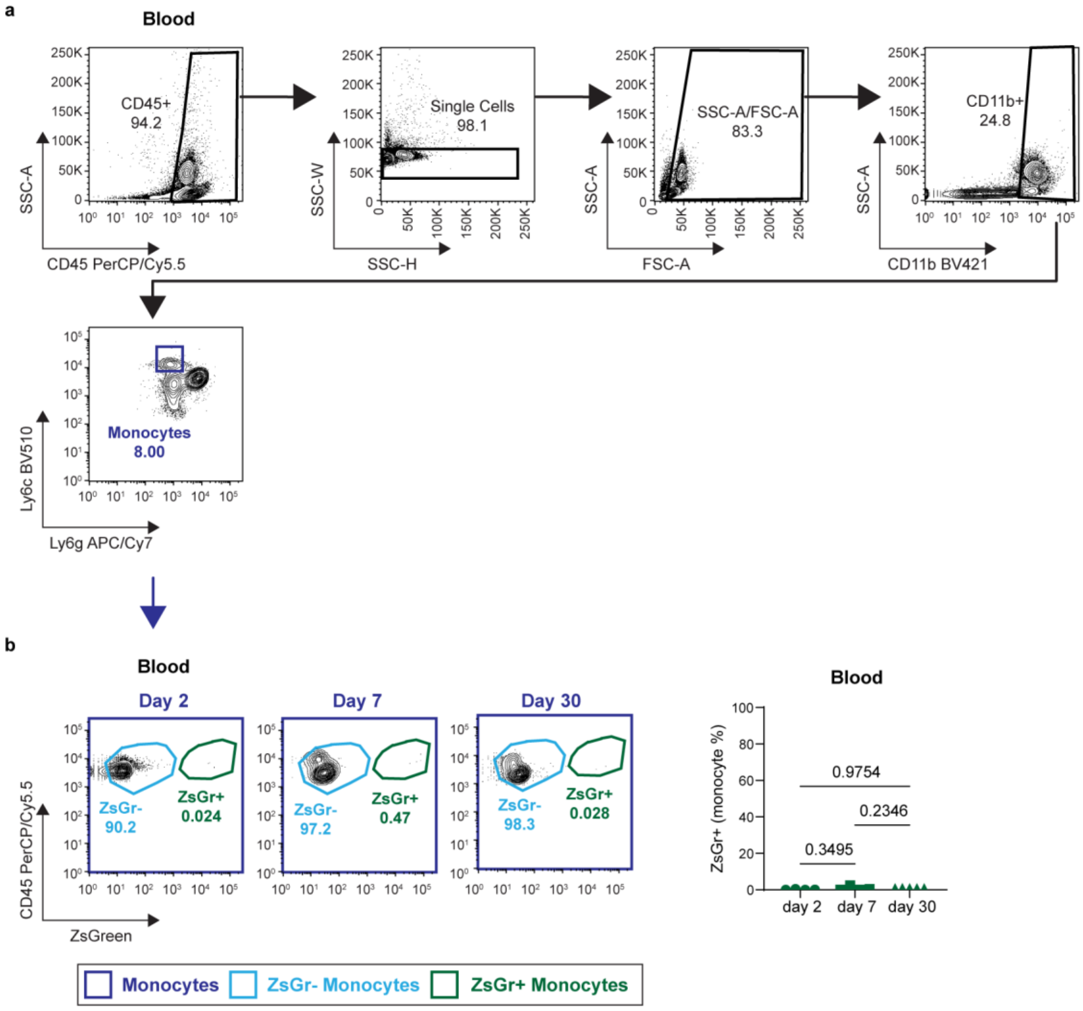

Supplementary Figure 9. ZsGr expression in the blood of Arg1ZsGr mice after heart I/R. a, FACS gating strategy for the assessment of ZsGr$^-$ and ZsGr$^+$ cells in the blood of Arg1ZsGr mice after heart I/R. **b,** FACS analysis of ZsGr- and ZsGr+ cells from the monocytes gate in a. Quantification shows the amount of ZsGr$^+$ cells as a percentage of the monocyte gate. N = 4 vs 4 vs 5 (each data point represents an individual mouse). P-values are determined using the ordinary one-way ANOVA using Tukey's multiple comparisons test.

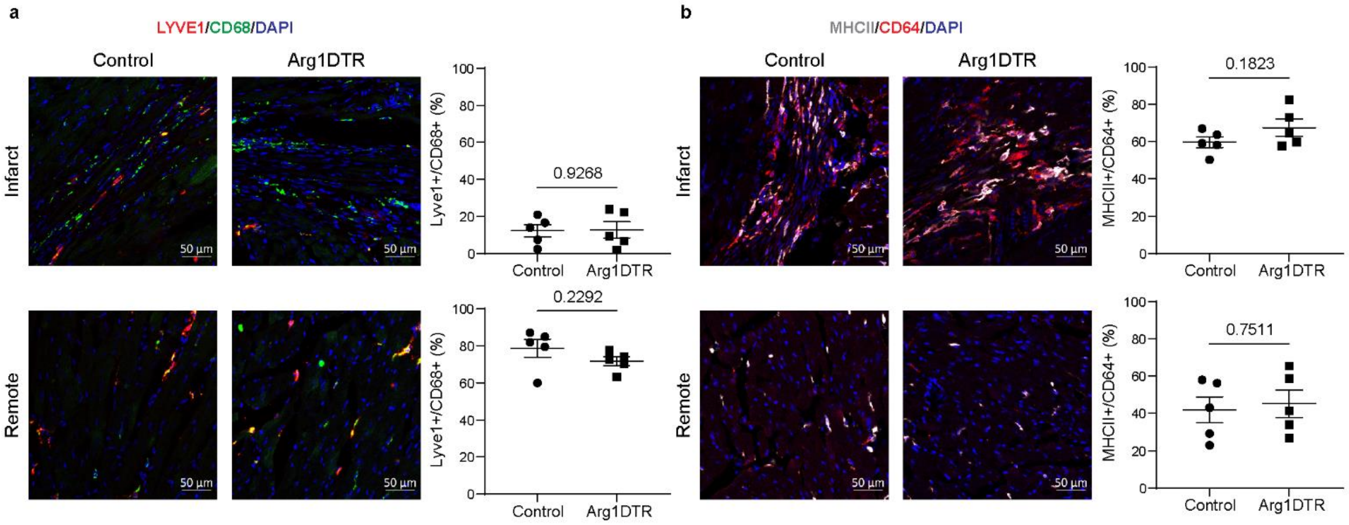

Supplementary Figure 10. Histological and IHC analysis of Arg1DTR mice 4 weeks after I/R. a, 20x confocal images of LYVE1 (red), CD68 (green), and DAPI (blue) IHC staining within the infarct and remote zones 4 weeks after I/R in Control and Arg1DTR mice. Quantification displayed as the percentage of $LYVE1^{+}CD68^{+}$ cells out of total $CD68^{+}$ cells per 20x field. **b,** 20x confocal images of MHCII (white), CD64 (red), and DAPI (blue) IHC staining with the infarct and remote zones 4 weeks after I/R in Control and Arg1DTR mice. Quantification displayed as the percentage of $MHCII^{+}CD64^{+}$ cells out of total $CD64^{+}$ cells per 20x field. N = 5 (each data point represents an individual mouse). P-values are determined using a two tailed t-test assuming equal variance.
